# Stochastic parameter search for events

**DOI:** 10.1186/s12918-014-0126-y

**Published:** 2014-11-08

**Authors:** Min K Roh, Philip Eckhoff

**Affiliations:** Numerical Methods, Institute for Disease Modeling, 1575 132 Ave. NE, Bellevue, 98005 USA

**Keywords:** Stochastic simulation, Parameter estimation, Rare event, Optimization

## Abstract

**Background:**

With recent increase in affordability and accessibility of high-performance computing (HPC), the use of large stochastic models has become increasingly popular for its ability to accurately mimic the behavior of the represented biochemical system. One important application of such models is to predict parameter configurations that yield an event of scientific significance. Due to the high computational requirements of Monte Carlo simulations and dimensionality of parameter space, brute force search is computationally infeasible for most large models.

**Results:**

We have developed a novel parameter estimation algorithm—Stochastic Parameter Search for Events (SParSE)—that automatically computes parameter configurations for propagating the system to produce an event of interest at a user-specified success rate and error tolerance. Our method is highly automated and parallelizable. In addition, computational complexity does not scale linearly with the number of unknown parameters; all reaction rate parameters are updated concurrently at the end of each iteration in SParSE. We apply SParSE to three systems of increasing complexity: birth-death, reversible isomerization, and Susceptible-Infectious-Recovered-Susceptible (SIRS) disease transmission. Our results demonstrate that SParSE substantially accelerates computation of the parametric solution hyperplane compared to uniform random search. We also show that the novel heuristic for handling over-perturbing parameter sets enables SParSE to compute biasing parameters for a class of rare events that is not amenable to current algorithms that are based on importance sampling.

**Conclusions:**

SParSE provides a novel, efficient, event-oriented parameter estimation method for computing parametric configurations that can be readily applied to any stochastic systems obeying chemical master equation (CME). Its usability and utility do not diminish with large systems as the algorithmic complexity for a given system is independent of the number of unknown reaction rate parameters.

**Electronic supplementary material:**

The online version of this article (doi:10.1186/s12918-014-0126-y) contains supplementary material, which is available to authorized users.

## Background

Stochastic modeling of biochemical and ecological systems has become increasingly popular due to its ability to represent system dynamics correctly at a detailed level, especially when species are present at low population. Deterministic models, on the other hand, are easier to analyze, yet they may fail to capture even the average behavior when the represented system exhibits nonlinearity [1] or is near extinction. Recent advancements in cloud computing platforms [2,3] and GPU computing [4-7] have significantly increased the affordability of computational resources. This enables development and use of stochastic algorithms that would have been deemed computationally infeasible in the past. However, there is still a void in stochastic methods that can answer scientifically interesting questions. One such application is in determining reaction rate configurations that yield an event of interest with a set success probability. Most parameter estimation algorithms in stochastic chemical kinetics setting take time-series data as an input and compute a set of reaction rate parameters that most closely reproduce the data. Methods used to determine these reaction rate parameters include maximum likelihood ratio [8-10], gradient decent [11], and moment closure [12]. While these algorithms are useful in its own right, scientists are often interested in knowing all parameter combinations that yield a specific event of interest. For gene regulatory models, knowledge of all pathways to achieve a specific event, such as bistable transition of *lac* operon in *E. coli* [13-15], may be used to guide laboratory experiments. In epidemiological models, all intervention parameter combinations that achieve eradication can be combined with econometrics in computing the most cost-effective strategy for eradicating a disease [16]. To authors’ knowledge, no algorithm has been developed in stochastic chemical kinetics setting that computes such parameter combinations.

In this paper, we present Stochastic Parameter Search for Events (SParSE) that finds a parametric hyperplane of reaction rates conferring a user-specified event with prescribed success rate and error tolerance. Our algorithm is robust in that it accurately computes the solution hyperplane for low probability events as well as high probability events. It is also trivial to parallelize the algorithm; initial parameter sets do not need to communicate with each other to find the direction to the unknown solution hyperplane. Once the algorithm finds a point in the solution hyperplane, the ratio between the initial and final rates can be used as the biasing parameters by the doubly weighted stochastic simulation algorithm (dwSSA) [17] to compute the probability of observing the target event with its success rate under the original system description. This allows calculation of the target event probabilities under the original parameters as a powerful side benefit of the algorithm. Lastly, the SParSE runtime per parameter sample is of the same order as that of the stochastic simulation algorithm (SSA), *i.e.*, the algorithm complexity is independent of the number of unknown parameters for a given system. This is achieved by combining a novel modification of dwSSA [17], Rubinstein’s cross-entropy method [18], and exponential interpolation of biasing parameters. This feature provides substantial benefits when searching multi-dimensional parameter space.

## Methods

### Doubly weighted stochastic simulation

We begin with a brief review of the dwSSA; detailed derivation and applications can be found in Daigle *et al*. [17]. Throughout this paper we assume a well-stirred system at constant temperature with *N* species *S*_1_,…,*S*_*N*_ and *M* reactions *R*_1_,…,*R*_*M*_. The state of the system at time *t* is represented as **X**(*t*)=[*X*_1_,…,*X*_*N*_], where *X*_*i*_ is the population of species *S*_*i*_. Using the “direct method” implementation of Gillespie’s Stochastic Simulation Algorithm (SSA) [19], the system moves forward in time by sequentially firing one of *R*_*j*_, *j*∈{1,…,*M*} reactions, whose propensity at time *t* is *a*_*j*_(**X**(*t*)) and its sum $a_{0} = \sum _{j=1}^{M} a_{j}(\mathbf {X}(t))$. Here, the next reaction is chosen with a categorical random variable *j*^′^ and the time to the next reaction with an exponential random variable *τ*. We also assume that all trajectories are run until the smaller of the final simulation time *t*_*f*_ and the first time  is observed, where  is the event of interest. Denoting the stopping time of a trajectory as , the probability of a complete trajectory $J = \left (t_{1}, j^{\prime }_{1},\dots,t_{N_{\mathcal {T}}}, j^{\prime }_{N_{\mathcal {T}}}\right)$ given **X**(0)=**x**_0_ under SSA is as follows:

(1) $$\begin{array}{@{}rcl@{}} \mathrm{P}_{SSA}\left(\mathbf{J}\right) & = & \prod\limits_{i=1}^{N_{\mathcal T}} \left[ a_{0}(\mathbf{X}(t_{i})) e^{-a_{0}(\mathbf{X}(t_{i})) \tau_{i}} d \tau_{i} \times \frac{a_{j^{\prime}_{i}}(\mathbf{X}(t_{i}))}{a_{0}(\mathbf{X}(t_{i}))} \right]  \\ & = & \prod\limits_{i=1}^{N_{\mathcal{T}}} \left[ a_{j^{\prime}_{i}}(\mathbf{X}(t_{i})) e^{-a_{0}(\mathbf{X}(t_{i})) \tau_{i}} d \tau_{i} \right], \end{array} $$

with $t_{i} \equiv \sum _{j=1}^{i} \tau _{j}$ and $N_{\mathcal T}$ the total number of reactions fired in $[\!0,\mathcal {T}]$.

The dwSSA uses predilection functions to increase the number of trajectories that reach :

(2) $$ b_{j}(\mathbf{X}(t)) \equiv \gamma_{j} a_{j}(\mathbf{X}(t)), \quad b_{0}(\mathbf{X}(t)) = \sum\limits_{j=1}^{M} b_{j}(\mathbf{X}(t)),  $$

where $\gamma _{j} \in \mathbb {R^{+}}$ is a biasing parameter for *R*_*j*_. The probability of the same trajectory **J** under the dwSSA is then given by

(3) $$\begin{array}{@{}rcl@{}} \mathrm{P}_{dwSSA}(\mathbf{J}) & = & \prod\limits_{i=1}^{N_{\mathcal T}} \left[ b_{0}(\mathbf{X}(t_{i})) e^{-b_{0}(\mathbf{X}(t_{i})) \tau_{i}} d \tau_{i} \times \frac{b_{j^{\prime}_{i}}(\mathbf{X}(t_{i}))}{b_{0}(\mathbf{X}(t_{i}))} \right]  \\ & = & \prod\limits_{i=1}^{N_{\mathcal T}} \left[ b_{j^{\prime}_{i}}(\mathbf{X}(t_{i})) e^{-b_{0}(\mathbf{X}(t_{i})) \tau_{i}} d \tau_{i} \right], \end{array} $$

and the bias incurred by using the predilection function is corrected with

(4) $${} {\fontsize{9pt}{9.6pt}\selectfont{\begin{aligned} W_{dwSSA}\left(\mathbf{J}\right) & = \prod\limits_{i=1}^{N_{\mathcal T}} \left[ \frac{a_{j^{\prime}_{i}}(\mathbf{X}(t_{i})) e^{-a_{0}(\mathbf{X}(t_{i})) \tau_{i}}}{b_{j^{\prime}_{i}}(\mathbf{X}(t_{i})) e^{-b_{0}(\mathbf{X}(t_{i})) \tau_{i}}} \right] \\ & = \prod\limits_{i=1}^{N_{\mathcal{T}}} \left[ \exp{\left\{ \left(b_{0}(\mathbf{X}(t_{i})) - a_{0}(\mathbf{X}(t_{i})) \right) \tau_{i} \right\}} \times (\gamma_{j^{\prime}_{i}})^{-1} \right]. \end{aligned}}}  $$

It is straightforward to confirm that the product of (3) and (4) equals (1).

The Monte Carlo estimator for $p_{\textit {dwSSA}}(\mathbf {x}_{0}, \mathcal E; t)$, the probability that the system reaches  by time *t* given the initial state **x**_0_, is

(5) $$ \hat{p}_{dwSSA}(\mathbf{x}_{0}, \mathcal E; t) = \frac{1}{N} \sum\limits_{i=1}^{N} \left[ I_{\{\mathbf{J}_{i} \cap \mathcal E\}} W_{dwSSA}(\mathbf{J}_{i}) \right],  $$

where *N* is the total number of trajectories, **J**_*i*_ represents the *i*^th^ simulated dwSSA trajectory, and $I_{\{\mathbf {J}_{i} \cap \mathcal {E}\}}$ takes a value of 1 if  is visited by **J**_*i*_ and 0 otherwise. The quantity in (5) can be interpreted as the weighted average of successful trajectories, *i.e.*, trajectories reaching , where the weight is computed according to (4). A good set of biasing parameters would yield successful trajectories with weights close to the true probability and thus reduce variance in the probability estimator. The dwSSA computes low variance biasing parameters by minimizing cross entropy using a modified version of Rubinstein’s multilevel cross-entropy method [17,18]. The advantage of minimizing cross entropy over minimizing variance is that the former yields biasing parameters with a closed-form solution for (2) where the latter does not. Having a closed-form solution is of practical necessity, as the alternative would be to solve a large set of nonlinear equations, significantly decreasing the efficiency of the algorithm if not making the simulation infeasible.

Following derivations presented in Daigle *et al*. [17], the dwSSA biasing parameter for *R*_*j*_ is computed as

(6) $${} {\fontsize{8.8pt}{9.6pt}\selectfont{\begin{aligned} \hat{\gamma}_{j}^{(l)} \,=\,\frac{\sum_{i}^{\prime} \left(W_{dwSSA}\left(\mathbf{J}_{i}^{(l-1)}; \hat{\boldsymbol{\gamma}}^{(l-1)}\right) \times n_{ij} \right)}{\sum_{i}^{\prime} \left(W_{dwSSA}\!\left(\mathbf{J}_{i}^{(l-1)}; \hat{\boldsymbol{\gamma}}^{(l-1)}\right) \!\times\! \sum_{k=1}^{N_{{\mathcal{T}}_{k}}}\! \left[ a_{j}\left(\mathbf{X}_{i}^{(l-1)}(t_{ik})\right) \!\tau_{ik} \right] \right)}, \end{aligned}}}  $$

where *n*_*ij*_ is the total number of times reaction *j* fires in the *i*^th^ trajectory, $\sum _{i}^{\prime }$ iterates only over trajectories reaching , and *l* is the stage index in multilevel cross-entropy method. Computation for $\hat {\boldsymbol {\gamma }}^{(l)}$ terminates when intermediate rare event reaches , at which point we set $\hat {\boldsymbol {\gamma }}^{(l)} \equiv \hat {\boldsymbol {\gamma }}^{*}$.

The objective of the dwSSA necessitates computation of the likelihood ratio (4) as its probability estimator is with respect to the initial reaction rates ***k***^(0)^. On the other hand, the objective of SParSE is to compute a set of reaction rates $\boldsymbol {k}^{*} \in \mathbb {R}^{M+}$ such that

(7) $$ \left|\mathcal{P_{E}} - \frac{1}{N} \sum_{i=1}^{N} \left[ I_{\{f_{i}(\textbf{x}(t|\boldsymbol k^{*})) \cap \mathcal E\}} \right]\right| \leq \epsilon_{\mathcal{P_{E}}},  $$

where $\mathcal {P_{E}}$ is the desired probability of observing  by time *t*, $ I_{\{f_{i}(\textbf {x}(t|\boldsymbol {k}^{*})) \cap \mathcal {E}\}}$ an indicator function for observing  during *i*^th^ trajectory, and $\epsilon _{\mathcal {P_{E}}}$ a user-specified absolute error tolerance on $\mathcal {P_{E}}$. Unlike the dwSSA where the biasing parameters are updated each level of the multilevel cross-entropy method according to (6), in SParSE reaction rates are updated instead. We note that it is possible to use the dwSSA Monte Carlo estimator (5) in (7) and update ***γ***^(*l*)^ instead of ***k***^(*l*)^. However unless ***k***^(0)^ is sufficiently close to ***k***^∗^, the likelihood ratio (4) may become extremely small, *i.e.*, degenerate, and updating reaction rates avoids this problem. We discuss the criteria for updating ***k*** in the following section.

### Multilevel cross-entropy method

The modification of multilevel cross-entropy method in SParSE is similar to that of Ref [17]. However, there are three major differences between the multilevel cross-entropy method employed by dwSSA and by SParSE: (i) dwSSA only computes a single intermediate event *ξ*^(*l*)^ and the corresponding set of biasing parameters ***γ***^(*l*)^ while SParSE may compute multiple such quantities, (ii) SParSE can calculate biasing parameters for initial reaction rates that either over- or under-perturb the system with respect to $\mathcal {P}_{\mathcal {E}}$. For an over-perturbed system, it applies inverse biasing to reaction rates to convert  from a “sure event” to a “rarer event”, and (iii) dwSSA updates biasing parameters ***γ***^(*l*)^ while SParSE updates reaction rates ***k***^(*l*)^. The following subsection explains the first two differences and highlights how SParSE achieves the same time complexity as dwSSA for computing quantities in (i). The next subsection focuses on (iii) and its effect on simulation details.

#### Concurrent computation of multiple intermediate events and biasing parameters

In dwSSA, *N* trajectories in level *l* of multilevel cross-entropy method are run until either the final simulation time *t*_*final*_ or the first occurrence of *ξ*^(*l*)^, where *ξ*^(*l*)^ is an intermediate rare event chosen by the top ⌈*ρN*⌉ trajectories that evolve farthest in the direction of . Typical value of *ρ* used in dwSSA is 0.01 for *N*=10^5^, although any value *ρ*∈(0,1) can be used in theory [17]. The role of *ρ* can be thought as a knob that controls the tradeoff between the speed of convergence to  and accuracy in $\hat {\boldsymbol {\gamma }}^{*}$. For *ρ*^′^<*ρ*, we get $|\mathcal E - \xi ^{(l')}| \leq |\mathcal {E} - \xi ^{(l)} |$, thus smaller values for *ρ* can potentially drive the system toward  faster. However the number of trajectories reaching $\xi ^{(l^{\prime })}$ is less than the number of trajectories reaching *ξ*^(*l*)^ since ⌈*ρ*^′^*N*⌉<⌈*ρN*⌉. Having fewer data to compute $\hat {\boldsymbol {\gamma }}^{(l)}$ reduces the confidence on the estimate, therefore it is advised to keep ⌈*ρN*⌉ above a set threshold (*e.g.*, 200) in practice. On the other hand, larger value of *ρ* (*e.g.*, *ρ*>0.3) implies a less selective intermediate rare event. The resulting biasing parameters may not push the system closer to , causing a failure in convergence to the target event. In our experience, *ρ*<0.2 and ⌈*ρN*⌉>100 yield both reliable computation of biasing parameters and acceptable convergence to .

In order to determine *ξ*^(*l*)^, it is necessary to determine the direction of bias in addition to *ρ*. This is done by grouping the initial state **x**_0_ into two categories according to its distance with respect to the event of interest:

(8) $$ \phi_{\text{type}} = \left\{\begin{aligned} &1 \qquad\,\, \text{if}\, \, f\, \, \left(\textbf{x}\left(t_{0}\right)\right) \leq \mathcal{E} \\ &-1 \quad \text{otherwise}, \end{aligned}\right.  $$

where *f*(**x**(*t*)) is an event function. Two requirements for *f*(**x**(*t*)) are that it takes **x**(*t*) as an input and can be used to evaluate the distance between the current state and  (*i.e.*, it can be used to compute extreme values of each trajectory to determine the next intermediate event). The value of *ϕ*_type_ indicates *initial* position of **x**(*t*) with respect to  at *t*=0. When *ϕ*_type_ is equal to 1, maximum value of *f*(**x**(*t*)) in each trajectory is recorded, and *N* such values are sorted in descending order at the end of the simulation. The reasoning for this is that since $f\left (\textbf {x}\left (t_{0}\right)\right) \leq \mathcal {E}$, we need to encourage higher *f*(**x**(*t*)) values to get closer to . Similarly, minimum *f*(**x**(*t*)) values are recorded and sorted in ascending order when *ϕ*_type_ is -1. For convenience we refer to the sorted array of extreme *f*(**x**(*t*)) values as **v**_***k***_, where ***k*** is the reaction rates used to generate **x**(*t*).

We now define a SParSE probability estimator for :

(9) $$ \hat{p}_{SParSE}(\mathbf{x}_{0}, \boldsymbol{k}, \mathcal{E}; t) = \frac{1}{N} \sum\limits_{i=1}^{N} \left[ I_{\{\,f_{i}(\textbf{x}(t|\boldsymbol{k})) \cap \mathcal{E}\}} \right],  $$

where *f*_*i*_(**x**(*t*|***k***)) are event function evaluated with *i*^*th*^ SSA trajectory generated with reaction rates ***k*** and $I_{\{f_{i}(\textbf {x}(t|\boldsymbol {k})) \cap \mathcal {E}\}}$ takes a value of 1 if *f*_*i*_(**x**(*t*|***k***)) contains  and 0 otherwise. Once *N* trajectories are simulated, we can expect one of the following outcomes: (a) the inequality in (7) is satisfied, (b) $\hat {p}_{\textit {SParSE}}(\mathbf {x}_{0}, \boldsymbol {k}, \mathcal {E}; t) < \left (\mathcal {P_{E}} - \epsilon _{\mathcal {P_{E}}}\right)$, or (c) $ \left (\mathcal {P_{E}} + \epsilon _{\mathcal {P_{E}}}\right) < \hat {p}_{\textit {SParSE}}(\mathbf {x}_{0}, \boldsymbol k, \mathcal {E}; t)$.

In the first case, SParSE exits and returns ***k*** as a successful output, i.e., a point in the solution hyperplane (***k***≡***k***^∗^). In the second case, we need to choose extreme values of *f*(**x**(*t*)) evolving furthest to , and we can view  as a “rare event” as in the dwSSA. Thus intermediate events and its respective biasing parameters are computed iteratively, each time taking the system closer to  with success rate $\mathcal {P_{E}}$. The last case corresponds to parameter sets that “over-perturb” the system, as  was reached with probability greater than $\mathcal {P_{E}}$. The method used to determine an intermediate event in the classical multilevel cross-entropy method cannot be applied here because we do not want trajectories that produce extreme values of *f*(**x**(*t*)). However, the information gathered from such trajectories can be used to quantify the behavior we *do not* want to observe. We achieve this by collecting the extreme values of *f*(**x**(*t*)) as in case (b), except that each SSA simulation is run until the final simulation time without stopping when  is observed. Once intermediate events are chosen and their corresponding biasing parameters are computed, we update *j*^*th*^ reaction rate *k*_*j*_ with 1/*γ*_*j*_. This inverse biasing discourages over-perturbation with respect to $\mathcal {P_{E}}$. Algorithms 2 and 3 in Appendix B of Additional file 1 contain pseudocode for (b) and (c), respectively.

Unlike the multilevel cross-entropy method used by dwSSA, where only one intermediate event is computed in each level of multilevel CE method, SParSE may choose multiple intermediate events. While it is not necessary to compute multiple intermediate events to reach the solution hyperplane, doing so greatly improves algorithm efficiency. The caveat here is that the efficiency gain occurs only when the biasing parameters for the multiple intermediate events are computed simultaneously. We start by describing the method SParSE uses to choose multiple intermediate events, all of which can be reached by *N* trajectories with sufficient frequency. This is attained by choosing multiple values for *ρ* that is a function of the distance to the desired target event probability $\mathcal {P_{E}}$. Denoting the distance as $\delta (\boldsymbol {k}) \equiv \mathcal {P_{E}} - \hat {p}_{\textit {SParSE}}(\boldsymbol {k})$, we have two different methods for choosing ***ρ***(*δ*): one for case (b) and the other for case (c). Handling of the two cases differ, as the result of inverse biasing is not as obvious as the normal biasing for case (b) is. In normal biasing strategy, updating intermediate reaction rates with a particular set of biasing parameters redistributes **v**_***k***_ such that the corresponding intermediate event becomes the mode. However, the inverse biasing operates on the heuristics of discouraging over-perturbation without knowing the exact effect on **v**_***k***_. Thus more conservative values for ***ρ*** are used in (11) to compensate for this difference. Lastly, we note that each of these cases can be detected by comparing the sign of *δ* to the value of *ϕ*_type_, where the equality represents case (b).

For sgn(*δ*(***k***))==*ϕ*_type_

(10) $$ \boldsymbol{\rho}(\delta) = \left\{\begin{aligned} &[\!0.005 \,\, 0.01] \qquad \text{if}\,\, 0.4 < |\delta| \\ &[\!0.01 \,\, 0.05 \,\, 0.1] \quad \text{if}\,\, 0.2 < |\delta| \leq 0.4\\ &[\!0.05 \,\, 0.1 \,\, 0.2] \quad\,\,\, \text{otherwise} \end{aligned}\right.  $$

For sgn(*δ*(***k***))≠*ϕ*_type_

(11) $$ \boldsymbol{\rho}(\delta) = \left\{\begin{aligned} &[\!0.01 \,\, 0.015] \qquad \text{if}\,\, 0.4 < |\delta| \\ &[\!0.05 \,\, 0.1 \,\, 0.15] \quad \text{if}\,\, 0.2 < |\delta| \leq 0.4\\ &[\!0.1 \,\, 0.15 \,\, 0.2] \quad\,\,\, \text{otherwise} \end{aligned}\right.  $$

As the distance to the target event decreases, SParSE selects less extreme values for intermediate events and vice versa. This reduces the risk of over- and under-perturbations. We note that the number of elements in ***ρ***(*δ*) does not necessarily correspond to the number of intermediate events chosen. For example, elements corresponding to positions ⌈0.005∗*N*⌉ and ⌈0.01∗*N*⌉ of **v**_***k***_ may be the same. We also note that a custom function can be used to compute ***ρ*** to better suit a specific system. However, the above default values work well for all examples presented in this paper. Lastly, *N* can be chosen as a function of min(***ρ***) and *c*, where *c* is the minimum number of data points desired to reliably compute ***γ***^(*l*)^, *i.e.*, *N*≥*c*/ min(***ρ***).

Once intermediate events are computed, they are sorted in ascending order of its probability, *i.e.*, *Prob*(*ξ*^(*l*,1)^) ≤⋯≤*Prob*(*ξ*^(*l*,*q*)^), where *q* is the number of unique intermediate events chosen at level *l*. We note that this sorting is done automatically if elements of ***ρ*** are sorted in ascending order, which (10, 11) are.

Now we describe how biasing parameters for all intermediate events are computed concurrently in a single ensemble of *N* simulations. In each simulation, we check for *ξ*^(*l*,*q*)^. If *ξ*^(*l*,*q*)^ is observed, the statistics gathered up to the time at which *ξ*^(*l*,*q*)^ was reached are used to compute ***γ***^(*l*,*q*)^. Then the trajectory continues its course of simulation, this time checking for *ξ*^(*l*,*q*−1)^ while keeping the cumulative statistics. This process repeats until the smaller of *t*_*final*_ and the time at which *ξ*^(*l*,1)^ is observed (*i.e.*, all intermediate events are observed). When *q*==1, this method is identical to the one used by dwSSA. Although a single trajectory runtime for *q*>1 is slightly longer than the runtime for *q*==1, the additional resources spent on concurrent computation is negligible compared to the savings of (*q*−1)·*N* simulations. We note that this process yields biasing parameter sets that are correlated because ***γ***^(*l*,*i*)^ is computed with a subset of data used to compute ***γ***^(*l*,*i*+1)^. However, this correlation does not affect the validity or the accuracy of the final output as only one set is selected at each level to update the reaction rates, the process for which we explain in the next section.

#### Updating intermediate reaction rates

SParSE propagates the system towards the solution hyperplane by iteratively updating reaction rates during the modified multilevel cross-entropy method. The update process requires choosing one set of biasing parameters from possibly many sets, where the set size is determined by the number of unique intermediate events. The current intermediate reaction rates are then multiplied element-wise by the chosen set to produce the next intermediate reaction rates. The criterion SParSE adopts is straightforward; at level *l* it chooses the biasing parameter set that, when multiplied to the current intermediate reaction rates, takes the system closest to  while preserving the sign of *δ*(***k***^(*g*)^),*g*=0,⋯,*l*.

Without loss of generality, we define ***k***^(cur)^ as the intermediate reaction rates at an arbitrary level *l*. In order to update the intermediate reaction rates for the next stage, we evaluate how each candidate biasing parameter set ***γ***^(*l*,·)^ performs with respect to the update criterion. We define ***k***^(int,*i*)^ as

(12) $$\begin{array}{@{}rcl@{}} k_{j}^{\text{(int},i)} = \left\{\begin{aligned} &k^{\text{(cur)}}_{j} \cdot \gamma_{j}^{(l,i)} \qquad \text{if}\,\, \text{sgn}{\left(\delta\left(\boldsymbol k^{\text{(cur)}}\right)\right)} == \phi_{\text{type}}\\ &k^{\text{(cur)}}_{j} \cdot 1/\gamma_{j}^{(l,i)} \quad \text{otherwise} \\ \end{aligned}\right., \ j \in \left\{1,\cdots,M\right\}, \ i \in \left\{1,\cdots,q\right\}, \end{array} $$

where *q* is the number of unique intermediate events. We recall that sgn(*δ*(***k***^(cur)^))≠*ϕ*_type_ corresponds to the case when $\left (\mathcal {P_{E}} + \epsilon _{\mathcal {P_{E}}}\right) < \hat {p}_{\textit {SParSE}}(\mathbf {x}_{0}, \boldsymbol k^{\text {(cur)}}, \mathcal {E}; t)$, which requires inverse biasing to reduce over-perturbation.

Starting with *i*=1, we compute $\hat {p}_{\textit {SParSE}}(\mathbf {x}_{0}, \boldsymbol k^{\text {(int,} i)}, \mathcal {E}; t)$. If $|\hat {p}_{\textit {SParSE}}\left (\boldsymbol k^{\text {(int,}i)}\right) \!\,-\,\! \mathcal {P_{E}}| \!\!\leq \!\! \epsilon _{\mathcal {P_{E}}}$, then the algorithm exits with ***k***^∗^=***k***^(int,*i*)^. Otherwise, we traverse through available sets of biasing parameters to find $\min \limits _{r}$$\left (\hat {p}_{\textit {SParSE}}(\boldsymbol k^{\text {(int,}r)}) < \left (\mathcal {P_{E}} - \epsilon _{\mathcal {P_{E}}}\right)\right)$ for sgn(*δ*(***k***^(cur)^))==*ϕ*_type_ and $\max \limits _{r} \left (\left (\mathcal {P_{E}} + \epsilon _{\mathcal {P_{E}}}\right) <\hat {p}_{\textit {SParSE}}(\boldsymbol {k}^{\text {(int,}r)})\right)$ for sgn(*δ*(***k***^(cur)^))≠*ϕ*_type_. Since ***ξ***^(*l*,·)^ are sorted in ascending order of its probability, ***k***^(int,*i*)^ is expected to produce more extreme *f*(**x**(*t*)) values than ***k***^(int,*i*+1)^. Thus it is not necessary to evaluate all possible $\hat {p}_{\textit {SParSE}}\left (\boldsymbol {k}^{\text {(int,}\cdot)}\right)$. For the case of under-perturbation we can stop the evaluation at the first occurrence of ***k***^(int,*i*)^ that satisfies the inequality and set ***k***^(l+1)^←***k***^(int,*i*)^. For the case of over-perturbation, however, we stop the simulation at the first occurrence of ***k***^(int,*i*)^ that *violates* the inequality and set ***k***^(l+1)^←***k***^(int,*i*−1)^.

It is possible that all candidate biasing parameter sets fail to satisfy the update criterion. The failure indicates $\mathcal {P_{E}}$ lies between $ \hat {p}_{\textit {SParSE}}\left (\boldsymbol {k}^{(l)}\right)$ and $\hat {p}_{\textit {SParSE}}\left (\boldsymbol {k}^{(\text {int},\cdot)}\right)$. Furthermore, this failure is a direct result of many-to-one relationship between $\boldsymbol {k} \in \mathbb {R}_{> 0}^{M}$ and $\xi \in \mathbb {R}$. Trajectories simulated with two different sets of reaction rates ***k*** and ***k***^′^=***k***+*ε* are likely to differ from each other, resulting in $\mathbf {v}_{\boldsymbol {k}} \neq \mathbf {v}_{\boldsymbol {k}^{\prime }}$. However, both **v**_***k***_ and $\mathbf v_{\boldsymbol {k}^{\prime }}$ may yield the same intermediate events, since they are determined solely by the value of the sorted array at positions ⌈***ρ****N*⌉. Despite the identical ***ξ***, SParSE estimates computed with ***k*** and ***k***^′^ will differ if the proportion of occurrences of  is not the same in the two arrays.

In summary, the modified multilevel cross-entropy method for SParSE comprises of 3 steps. First we determine intermediate events for the current reaction rates using the SSA. We then employ dwSSA simulations to compute biasing parameters for each of the intermediate events. Lastly we follow steps described in this section to choose one set of biasing parameters to update reaction rates for the next iteration. This process repeats until either ***k***^∗^ is found or until intermediate reaction rates cannot be updated any more. For computational efficiency, we can combine the first and the last steps by computing $\mathbf {v}_{\boldsymbol {k}}^{(\text {int},i)}$ at the same time as computing $\hat {p}_{\textit {SParSE}}\left (\boldsymbol {k}^{(\text {int},i)}\right)$. We discard $\mathbf {v}_{\boldsymbol {k}}^{(\text {int},i)}$ if the estimate does not satisfy the required inequality or if ***k***^(int,*i*)^ is not the best candidate for the next intermediate reaction rates.

Lastly we point out that the original dwSSA formula (6) for computing ***γ***^(*l*)^ requires computation of a trajectory weight, which is the product of the likelihood ratio between the propensity function and the predilection function. We recall that the predilection function in the multilevel cross-entropy method used by dwSSA is characterized with the biasing parameters from level (*l*−1). However, we do not need to compute the trajectory weight in SParSE because its objective is to find a new set of reaction rates that confer the event specifications regardless of ***k***^(0)^, which is used only as a starting position in the path to find ***k***^∗^. The intermediate reaction rates in level *l* of SParSE reflect cumulative amount of bias applied to the original system up to stage *l*−1 as quantified in (12). Thus the propensity function at level *l* does not require additional biasing. This leads to using SSA to determine intermediate events and dwSSA to compute ***γ***^(*l*)^ with $\gamma ^{(l-1)}_{j} = 1, \ j \in \{1,\cdots,M\}$. Therefore the formula (6) for SParSE simplifies to

(13) $$ \hat{\gamma}_{j}^{(l)} = \frac{\sum_{i}^{\prime} n_{ij}}{\sum_{i}^{\prime} \sum_{k=1}^{N_{\mathcal{T}_{k}}} \left[ a_{j}\left(\mathbf{X}_{i}^{\left(l-1\right)}\left(t_{ik}\right)\right) \tau_{ik} \right]} \;.  $$

### Exponential interpolation of biasing parameters

Iteratively updating intermediate reaction rates via the modified multilevel cross-entropy method described in the previous section may not find ***k*** that satisfies (7). Possible reasons for the failure include poor choice of ***ρ***, insufficient *N*, and nonexistence of candidate intermediate reaction rates that satisfy the update criterion. The first two aligned can lead to slow convergence to , especially for systems near a deterministic regime or for simulations that demand high accuracy (*i.e.*, small values of $\epsilon _{\mathcal {P_{E}}})$. Setting a limit on the maximum number of iterations for the multilevel cross-entropy method can detect slow or non-converging reaction rates, and increasing *N* and/or modifying ***ρ*** will increase the rate of convergence in most aligned. The last phenomenon occurs when no suitable biasing parameters exist to update the reaction rates. Here we have $\left (\hat {p}(\boldsymbol {k}^{(u)}) +\epsilon _{\mathcal {P_{E}}}\right) < \mathcal {P_{E}} < \left (\hat {p}(\boldsymbol {k}^{(v)}) -\epsilon _{\mathcal {P_{E}}}\right)$, where $\hat {p}(\boldsymbol {k}^{\text {(u)}}) \,=\, \min \left (\hat {p}_{\textit {SParSE}}(\boldsymbol {k}^{\text {(cur)}}), \hat {p}_{\textit {SParSE}}(\boldsymbol {k}^{\text {(int,}i)})\right)$ and $\hat {p}(\boldsymbol {k}^{\text {(v)}}) \,=\, \max \left (\hat {p}_{\textit {SParSE}}(\boldsymbol {k}^{\text {(cur)}}), \hat {p}_{\textit {SParSE}}(\boldsymbol {k}^{\text {(int,}i)})\right)$, *i* =1,⋯,*q*. The target probability lies between the two estimates $\hat {p}(\boldsymbol {k}^{(u)})$ and $\hat {p}(\boldsymbol {k}^{(v)})$, and the multilevel cross-entropy method is unable to fine-tune intermediate reaction rates to achieve $\mathcal {P_{E}}$ within the specified error tolerance $\epsilon _{\mathcal {P_{E}}}$.

It is reasonable to assume that a linear combination of ***k***^(*u*)^ and ***k***^(*v*)^ may result in ***k***^∗^. A more sophisticated method for approximating ***k***^∗^ would be to fit an interpolant through past intermediate reaction rates. By making the following two assumptions, SParSE computes candidate biasing parameters such that when multiplied to ***k***^(0)^, they may satisfy (7).

#### **Assumption1**.

***k***^∗^ exists such that $k_{j}^{(u)} \leq k_{j}^{*} \leq k_{j}^{(v)}$ for $1 < k_{j}^{(v)} $ or $k_{j}^{(v)} \leq k_{j}^{*} \leq k_{j}^{(u)}$ for $k_{j}^{(v)} < 1$, *j*∈{1,⋯,*M*}.

#### **Assumption2**.

$k_{j}^{*}$ can be computed independently from $k_{h}^{*}$ for *j*≠*h*.

We note that a single dwSSA trajectory likelihood ratio at level *l* of the multilevel cross-entropy method is

(14) $$\begin{array}{*{20}l} {}L^{(l)}_{dwSSA}(\mathbf{J}) &= \prod\limits_{i=1}^{N_{\mathcal{T}}} \left[\vphantom{\left(\gamma_{j^{\prime}_{i}}^{(l-1)}\right)^{-1}}\exp{\left\{\left(a_{0}^{(l)}\left(\mathbf{X}(t_{i})\right) - a_{0}^{(l-1)}(\mathbf{X}(t_{i})) \right) \tau_{i} \right\}}\right.\\ &\quad\times\left. \left(\gamma_{j^{\prime}_{i}}^{(l-1)}\right)^{-1} \right], \end{array} $$

where $a_{0}^{(l-1)}(\mathbf {X}(t))$ and $a_{0}^{(l)}(\mathbf {X}(t))$ are the propensity sum of the trajectory **J** at time *t* generated with ***k***^(*l*−1)^ and ***k***^(*l*)^, respectively, and ***γ***^(*l*−1)^ is the biasing parameter used to update the intermediate reaction rates, *i.e.*, $k^{(l)}_{j} = k^{(l-1)}_{j} \times \gamma ^{(l-1)}_{j}, \ j \in \{1,\cdots,M\}$. The quantity inside the exponential term is a function of the system state, which is in turn a function of intermediate reaction rates. In order to compare SParSE estimates generated with different intermediate reaction rates, we rewrite them as a function of the initial reaction rates and normalized intermediate biasing parameters, *i.e.*, $k_{j}^{(\cdot)} = k_{j}^{(0)} \times \gamma _{j}^{(0,\cdot)}$, and $\gamma _{j}^{(0,0)} = 1$. The purpose here is to quantify the relationship between different values of ***γ***^(0,·)^ and its corresponding estimates $\hat {p}_{\textit {SParSE}}(\mathbf {x}_{0}, \boldsymbol {k}^{(\cdot)}, \mathcal {E}; t)$. Considering the form of the likelihood ratio in (14), a natural form for the interpolant is

(15) $$\begin{array}{@{}rcl@{}} g\left(\gamma_{j}^{(0,\cdot)}\right) = q_{j} \cdot \exp{\left\{ p_{j} \times \gamma_{j}^{(0,\cdot)} \right\} }, \end{array} $$

where *p*_*j*_ and *q*_*j*_ are constants and $\gamma _{j}^{(0,\cdot)}$ are normalized version of the intermediate biasing parameters used to compute past SParSE estimates. Output data used in constructing interpolants are the corresponding SParSE estimates $\hat {p}_{\textit {SParSE}}(\mathbf {x}_{0}, \boldsymbol {k}^{(\cdot)}, \mathcal {E}; t)$ multiplied by *N*, the total number of simulations. This particular form allows for fast solving of *p*_*j*_ and *q*_*j*_ with a first order polynomial curve fitting method. We first transform the data to logarithmic scale, compute for two coefficients in the first order polynomial, and then retransform the output with exponentiation. The reason for scaling the output data with *N* is to preserve as many significant digits as possible, as logarithmic y-scale is used to compute the polynomial coefficients. While other forms of interpolant may yield more accurate interpolation, (15) allows for fast computation while satisfying Assumption (1), as an exponential function is monotonic.

The number of past intermediate reaction rates available for interpolation varies by factors such as ***k***^(0)^, $\epsilon _{\mathcal {P_{E}}}$, and *N*. Although all past estimates can be used for interpolation, confining the number of data to *X* closest estimates of $\mathcal {P_{E}}$ (*e.g.X*=5) while having at least one estimate on either side of the target probability is recommended, as the accuracy of interpolation may degrade with estimates that are far from the target probability. Due to the construction of the algorithm, there exists at least one estimate on either side of $\mathcal {P_{E}}$ when the algorithm enters the exponential interpolation stage. However, we note that the total number of past intermediate reaction rates can be as few as 2. Once the values of *p*_*j*_ and *q*_*j*_ in (15) are determined for all *M* interpolants, SParSE executes the following steps to further increase the efficiency of simulation.

Step 1: For each *j*∈{1,⋯,*M*}, project $g(([{-2.0} \; {-1.0} {-0.5} \; 0.0 \;0.5 \;1.0 \; 2.0 ] \dot \epsilon _{\mathcal {P_{E}}} \,+\, \mathcal {P_{E}})\!\times \! N)$ onto the x axis of the interpolant to compute candidate biasing parameters $ \bar {\boldsymbol {\gamma }}_{j}^{(\cdot,s)}$, where the first element (·) in the superscript is the interpolation iteration index and *s*∈{1,⋯,7} is the index of the candidates.
Step 2: Compute candidate intermediate reaction rates $\bar {\boldsymbol {k}}^{(\cdot,s)}$, where $\bar {k}_{j}^{(\cdot,s)} = k_{j}^{(0)} \times \bar {\boldsymbol {\gamma }}_{j}^{(\cdot,s)}$
Step 3: Constrain $\bar {k}_{j}^{(\cdot,s)}$ to satisfy $k_{j}^{(u)} \leq \bar {k}_{j}^{(\cdot,s)} \leq k_{j}^{(v)}$ for $1 < k_{j}^{(v)}$ or $k_{j}^{(v)} \leq \bar {k}_{j}^{(\cdot,s)} \leq k_{j}^{(u)}$ for $k_{j}^{(v)} < 1$, *j*∈{1,⋯,*M*}, if necessary. Reverse the signs in inequalities for sgn(*δ*(***k***))≠*ϕ*_type_.

Starting with *s*=4, we compute $\hat {p}_{\textit {SParSE}}(\bar {\boldsymbol {k}}^{(\cdot,s)})$. We note that $\bar {\boldsymbol {k}}^{(\cdot,4)}$ corresponds to the reaction rates computed from projecting the exact target probability to the interpolating function. If executing Step 3 results in duplicate candidates, we eliminate the duplicate set(s) and assign the starting index to *s* such that $q_{j} \cdot \exp {\left \{ p_{j} \times \bar {\gamma }_{j}^{(\cdot,s)} \right \}} = \mathcal {P_{E}} N$.

If $\hat {p}_{\textit {SParSE}}(\bar {\boldsymbol {k}}^{(\cdot,s)})$ confers the target event probability within $\epsilon _{\mathcal {P_{E}}}$, SParSE exits with $\boldsymbol {k}^{*} \,=\, \bar {\boldsymbol {k}}^{(\cdot,s)}$. Otherwise, we compute the next estimate with $\bar {\boldsymbol {k}}^{(\cdot,s+1)}$ for $\hat {p}_{\textit {SParSE}}(\bar {\boldsymbol {k}}^{(\cdot,s)}) \!<\! \left (\mathcal {P_{E}}-\epsilon _{\mathcal {P_{E}}}\right)$, and $\bar {\boldsymbol {k}}^{(\cdot,s-1)}$ for $\left (\mathcal {P_{E}}+\epsilon _{\mathcal {P_{E}}}\right)\!<$$\hat {p}_{\textit {SParSE}}(\bar {\boldsymbol {k}}^{(\cdot,s)})$. The interpolation stage continues until either ***k***^∗^ is found or the end of candidate reaction rates is reached, at which point an additional interpolation may be executed with updated data. On a rare occasion, ***k***^∗^ lies between two candidate reaction rates without satisfying the error tolerance. This can lead to infinite loop of incrementing and decrementing *s* by 1 without converging to ***k***^∗^, but the cycle can easily be detected with a mask vector. SParSE implements one by creating a zero vector whose size is equal to the number of candidate biasing parameter sets. Every time a SParSE estimate is computed with candidate reaction rates at index *s*, we increment *s*^*th*^ position of the mask vector. Once the magnitude of any mask vector position becomes greater than 2, we conclude that ***k***^∗^ lies between two candidate biasing reaction rates. At this point, we have refined an upper and lower bound on ***k***^∗^, as all candidate biasing parameters computed satisfy the inequality in Assumption 1. Once the bounds are sufficiently small, an alternative to an additional interpolation is to take a weighted average of the two candidate reaction rates, where the weight is the distance between the SParSE estimate and $\mathcal {P_{E}}$. For the examples presented in the following section, we did not encounter any initial reaction rates that required such treatment.

## Results and discussion

We illustrate SParSE performance on the following three examples of increasing complexity: a birth-death process, a reversible isomerization process and a Susceptible-Infectious-Recovered-Susceptible (SIRS) disease transmission model. The first two examples were chosen to demonstrate the algorithm’s accuracy against the exact solution, which for these examples can be computed using the master equation or the infinitesimal generator matrix [1]. We then progress to a more complex SIRS model, which has no closed-form analytical solution. For each system, we analyze the SParSE performance on all possible combinations of $\mathcal {P_{E}} \in \{0.40, \ 0.60, \ 0.80\}$ and $\epsilon _{\mathcal {P_{E}}} \in \{0.01, \ 0.05, \ 0.10\}$, where $\mathcal {P_{E}}$ and $\epsilon _{\mathcal {P_{E}}}$ denote a desired probability for event  and its error tolerance, respectively. We then compare the result with that from comparable SSA simulations whose reaction rates are selected using uniform random sampling (URS). SParSE also employs URS but only to generate a number of initial reaction rates as a starting point, here set to 30. The number of simulations, *N*, used to estimate $\mathcal {P_{E}}$ per parameter sample is set to 5×10^4^ unless mentioned otherwise. We also test the robustness of SParSE by assessing its performance on a low probability event, $\mathcal {P_{E}} = 0.010$ and $\epsilon _{\mathcal {P_{E}}} = 0.001$ for the birth-death process, and a high probability event, $\mathcal {P_{E}} = 0.95$ and $\epsilon _{\mathcal {P_{E}}} = 0.005$ for the reversible isomerization process. The number of samples generated for SSA simulations with URS equals the total number of SParSE ensembles computed for a specific simulation scenario, which is the sum of the following quantities: the number of intermediate event computations, the number of estimates computed for each intermediate event, and the number of estimates computed in the exponential interpolation stage. Since the same number of trajectories is used for computing an intermediate event and a SParSE estimate, it is straightforward to compare the two strategies with computational fairness. For each simulation scenario, we provide four metrics on performance: the total number of SParSE estimates needed for all 30 initial parameter samples, the number of initial parameters that did not reach the solution hyperplane within 10 iterations of multilevel cross-entropy method or 3 iterations of exponential interpolation, the number of parameter sets that required interpolation in addition to the multilevel cross-entropy method, and the number of successful parameter sets generated by SSA simulations using URS for sampling reaction rates. Lastly, we provide movie files of SParSE ensemble simulations for two test scenarios: birth-death with $\mathcal {P_{E}} = 0.010$ and $\epsilon _{\mathcal {P_{E}}} = 0.001$ and SIRS with $\mathcal {P_{E}} = 0.40$ and $\epsilon _{\mathcal {P_{E}}} = 0.01$.

All computations were run on a desktop with Intel®; Xeon®; CPU E5-2680, 2.70 GHz processor with 8 cores and 32 GB of RAM. We utilized Matlab’s Parallel Computing Toolbox™ (PCT) and the Coder™. The PCT™ was used to simulate 8 SParSE ensembles in parallel while the Coder™ was used to convert frequently-used custom Matlab functions into low-level C functions for faster computation.

### Birth-death process

Our first example is a birth-death process.

$$ \begin{aligned} \emptyset & \overset{k_{1}}{\rightarrow} Y,& 1.0 \leq & k_{1} \leq 1.7 \\ Y & \overset{k_{2}}{\rightarrow} \emptyset, & 0.0125 \leq & k_{2} \leq 0.025 \end{aligned}  $$

with **x**_0_=[40] and the target event  being molecular count of *Y* reaching 80 before *t*=100. Table 1 summarizes the results for the 9 standard test aligned, where SParSE achieved 100% success rate in finding ***k***^∗^, a vector of reaction rates $\left [k_{1}^{*} \ k_{2}^{*}\right ]$ that confers desired $\mathcal {P_{E}}$ and $\epsilon _{\mathcal {P_{E}}}$, for 8 test aligned. For $\mathcal {P_{E}} = 0.60$ and $\epsilon _{\mathcal {P_{E}}} = 0.01$, two of thirty samples, $\boldsymbol {k}^{(0)}_{3} = \left [1.606 \; 0.0140\right ]$ and $\boldsymbol {k}^{(0)}_{27} = \left [1.684 \; 0.0148\right ]$ (subscript representing the index of initial reaction rates), failed to converge after three rounds of exponential interpolations. We discuss the details of the failure in Appendix C of Additional file 1.

**Table 1:** **Results of SParSE applied to the birth-death process**

$\boldsymbol{\mathcal {P_{E}}}$	$\epsilon _{\boldsymbol{\mathcal {P_{E}}}}$	**SParSE samples**	**Interpolations**	**Failures**	**Successful**
					**URS**
0.40	0.01	286 (35)	29 [1, 7, 19, 3]	0	3
	0.05	182 (33)	23 [7, 22, 1, 0]	0	12
	0.10	133 (29)	19 [11, 19, 0, 0]	0	11
0.60	0.01	319 (36)	30 [0, 3, 18, 9]	2	2
	0.05	164 (33)	26 [4, 25, 0, 1]	0	8
	0.10	120 (30)	18 [12, 18, 0, 0]	0	12
0.80	0.01	240 (51)	28 [2, 17, 10, 1]	0	2
	0.05	137 (43)	15 [15, 15, 0, 0]	0	6
	0.10	108 (37)	1 [29, 1, 0, 0]	0	12

The first column denotes the target probability, the second column absolute error tolerance, the third column the total number of SParSE samples computed for the 30 initial parameter sets with the number inside the parenthesis indicating the total number of intermediate event computations, the fourth the number of initial reaction rate parameter sets that required exponential interpolation, the fifth the number of initial sets that did not converge to the solution hyperplane, and the sixth the number of successful parameter sets generated with URS. For the fourth column, four numbers inside the bracket indicate the number of parameter sets that required 0, 1, 2, and 3 interpolations, respectively. *N* = 5 × 10^4^.

**Figure 1:**
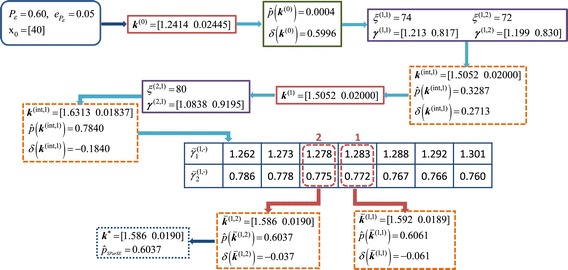
**Flow chart of SParSE simulation on the birth-death process with** $\boldsymbol{\mathcal {P}}_{\boldsymbol{\mathcal {E}}} = 0{.}60, \epsilon _{\boldsymbol{\mathcal {P}}_{\boldsymbol{\mathcal {E}}}} = 0{.}05, k^{(0)} = \left [ 1{.}2414 \; 0{.}02445\right ]$ **, and** ***N*** **=5×10** ^**4**^ **.** Arrows represent sequential steps taken by the algorithm.

**Figure 2:**
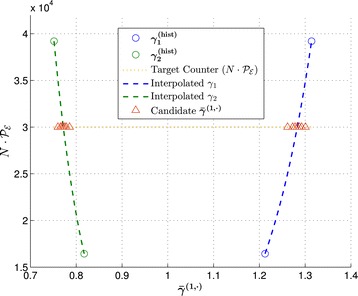
**Illustration of exponential interpolation for the birth-death process with** $\boldsymbol{\boldsymbol{\mathcal {P_{E}}}} = 0{.}60$ **,** $\epsilon _{\boldsymbol{\boldsymbol{\mathcal {P_{E}}}}} = 0{.}05, k^{(0)} = \, [\!1{.}2414 \; 0{.}02445]$ **, and** ***N*** **=5×10** ^**4**^ **.** Yellow horizontal dotted line is the desired number of successful trajectories, *i.e.*, $N \times \mathcal {P_{E}} = 3 \times 10^{4}$. Blue and green circles denote the past intermediate biasing parameters for *R*_1_ and *R*_2_, respectively, normalized with respect to ***k*** ^(0)^. Blue and green dashed lines are the interpolants constructed from the past intermediate basing parameters, and the red triangles are candidate biasing parameters computed according to Step 3 in Subsection *Updating intermediate reaction rates*.

**Figure 3:**
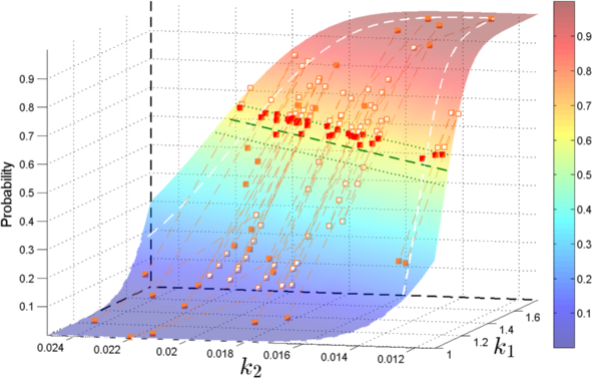
**Ensemble result for the birth-death process with** $\boldsymbol{\mathcal {P_{E}}} = 0{.}60, \ \epsilon _{\boldsymbol{\mathcal {P_{E}}}} = 0{.}05$ **, and** ***N*** **=5×10** ^**4**^ **.** SParSE required a total of 131 samples (30 initial, 71 intermediate, and 30 final). The green dashed line denotes the exact solution for $\mathcal {P_{E}} = 0.60$ and the green dotted lines represent ±0.05 absolute error tolerance band. Initial reaction rates are represented by orange squares, intermediate reaction rates by white squares, and ***k*** ^∗^s by red squares. Orange dashed lines connect any two subsequent reaction rates originated from the same ***k*** ^(0)^. White dashed lines represent the parameter ranges specified prior to simulation.

We picked one of 30 initial reaction rates for $\mathcal {P_{E}} = 0.60 \, \text {and} \, \epsilon _{\mathcal {P_{E}}} = 0.05$ to illustrate a complete progression of the algorithm. Figure 1 contains a flow chart of a SParSE run with $\boldsymbol {k}^{(0)}_{14} = \left [1.2414 \; 0.02445\right ]$. This particular set required two rounds of multilevel cross-entropy method and one exponential interpolation, which required computing two SParSE estimates (out of seven candidates) to reach the solution hyperplane $\left (\!\boldsymbol {k}^{*}_{(14)} = \left [1.586 \; 0.0190\right ]\!\right)$. Figure 2 shows an illustration of the interpolation results. We see from Table 1 that this particular scenario required 164 SParSE estimates (30 initial, 71 intermediate, and 30 final) in addition to 33 intermediate event computations in order to reach the solution hyperplane. An ensemble result is displayed in Figure 3, where the values of *z* axis are set to the probability of the target event, which is computed using *k*_1_ and *k*_2_ values defined by the data’s *x* and *y* coordinates, respectively. Together the figure shows the contour of the event probability surface for different values of *k*_1_ and *k*_2_. Despite a rapid change in the event probability around $\mathcal {P_{E}} = 0.60$, SParSE was able to find a point in the solution hyperplane for all 30 sets of initial reaction rates.

**Figure 4:**
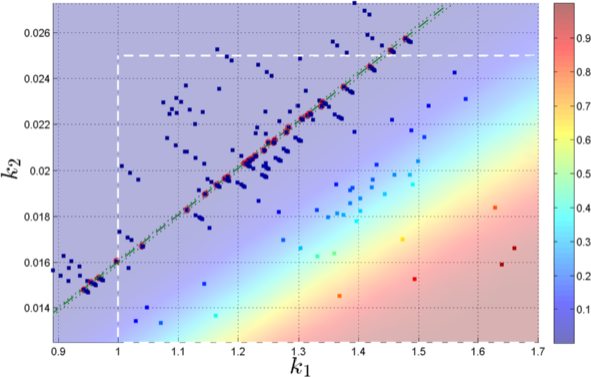
**SParSE ensemble result for the birth-death process with** $\boldsymbol{\mathcal {P_{E}}} = 0{.}010, \epsilon _{\boldsymbol{\mathcal {P_{E}}}} = 0{.}001$ **, and** ***N*** **=2×10** ^**5**^ **.** SParSE required a total of 215 samples (30 initial, 155 intermediate, and 30 final). The green dashed line denotes the exact solution for $\mathcal {P_{E}} = 0.010$ and the green dotted lines ±0.001 absolute error tolerance band. Final reaction rates ***k*** ^∗^ are encircled in red. White dashed lines represent the parameter ranges specified prior to simulation.

**Figure 5:**
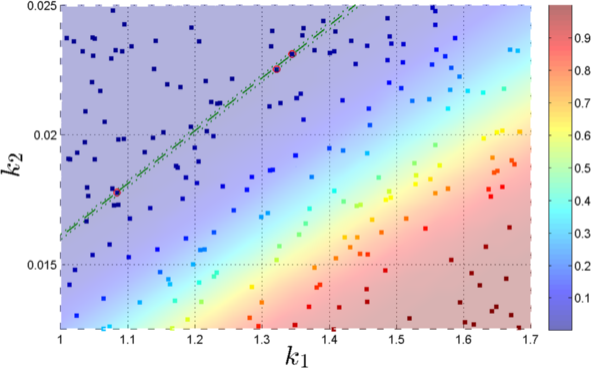
**SSA-URS ensemble result for the birth-death process with** $\boldsymbol{\mathcal {P_{E}}} = 0{.}010, \ \epsilon _{\boldsymbol{\mathcal {P_{E}}}} = 0{.}001$ **, and** ***N*** **=2×10** ^**5**^ **.** Color of each square represents $\mathcal {P_{E}}$ given its *k*_1_ and *k*_2_ values according to the color bar given on the right. Legend identities match those of Figure 4.

Next we illustrate the robustness of SParSE by choosing a very small target probability $\mathcal {P_{E}} = 0.010$ and $\epsilon _{\mathcal {P_{E}}} = 0.001$ (animated illustration of SParSE simulations for this scenario is provided as Additional file 2). For this problem, we increased *N* to 2×10^5^ to reduce the relative uncertainty in the estimate [20]. Table 2 summarizes the results. We see that all 30 initial sets of reaction rates successfully converged to the solution hyperplane while SSA-URS yielded only 3 successful samples. Figure 4 displays all 215 SParSE samples (30 initial, 155 intermediate, and 30 final) for this scenario. Figure 5 displays result of the same simulation scenario using SSA-URS, except that it contains 36 additional data to accommodate the total number of intermediate event computations SParSE required. We note that the parameter ranges shown in Figure 4 differ from that in Figure 5, whose data obey parametric constraints specified in the model description (*i.e.*, 1.0≤*k*_1_≤1.7 and 0.0125≤*k*_2_≤0.025). These constraints are shown as white dashed lines in Figure 4. The reason Figure 4 contains data outside the perimeter of white dashed lines is that our implementation of SParSE does not utilize the parametric constraints other than to generate initial sets of reaction rates. Changing the implementation of SParSE to enforce the parametric constraints throughout the simulation requires the user to provide a parametric region that contains the solution hyperplane. In this alternate implementation, if the solution hyperplane does not exist within the user-specified region, all computations are wasted. The current implementation allows for computation of the solution hyperplane regardless of its location while exploiting the user’s knowledge in generating initial reaction rates.

**Table 2:** **Results of SParSE applied to the birth-death process**

$\boldsymbol{\mathcal {P_{E}}}$	$\epsilon _{\boldsymbol{\mathcal {P_{E}}}}$	**SParSE samples**	**Interpolations**	**Failures**	**Successful**
					**URS**
0.010	0.001	251 (36)	27 [3, 17, 9, 1]	0	3

The column identities match those of Table 1. *N* = 2 × 10^5^.

### Reversible isomerization process

**Figure 6:**
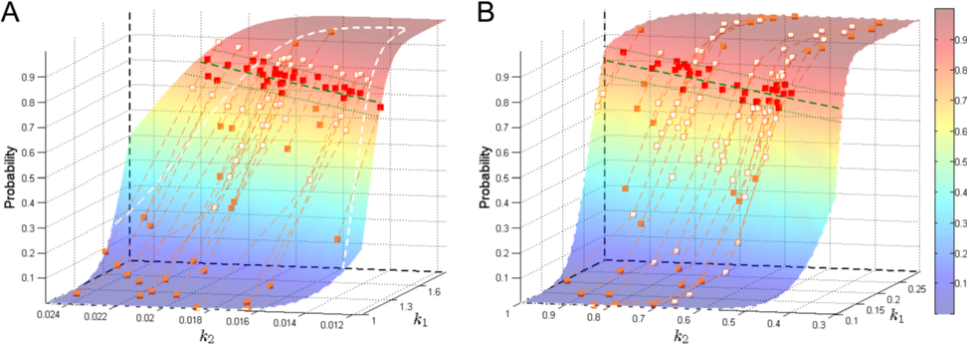
**SParSE ensemble result comparison for** $\boldsymbol{\mathcal {P_{E}}} = 0{.}80, \ \epsilon _{\boldsymbol{\mathcal {P_{E}}}} = 0{.}05$ **, and** ***N*** **=5×10** ^**4**^ **.** **A** and **B** correspond to the birth-death process and the reversible isomerization process, respectively. Legend identities match those of Figure 4.

Our next example concerns a reversible isomerization process, where two conformational isomers *A* and *B* are interconverted by rotation about single bonds:

$$\begin{aligned} A & \overset{k_{1}}{\rightarrow} B,& 0.1 \leq & k_{1} \leq 0.3 \\ B & \overset{k_{2}}{\rightarrow} A, & 0.3 \leq & k_{2} \leq 1.0, \end{aligned} $$

with **x**_0_= [ 100 0], *i.e.,* all molecules are initially in *A* form. The target event is set to population of *B* reaching 30 before *t*=10. Table 3 summarizes the results from 9 standard test scenarios, all of which attained 100% convergence to the solution hyperplane. We see that the total number of SParSE samples required for $\mathcal {P_{E}} \in \{0.40, \ 0.60\}$ is comparable between the birth-death and the reversible isomerization processes. However, the latter required considerably more number of samples for $\mathcal {P_{E}} = 0.80$. This is due to the difference in the contour of target event probability surface between the two processes. Figure 6 compares ensemble results between the two processes for $\mathcal {P_{E}} = 0.80$ and $\epsilon _{\mathcal {P_{E}}} = 0.05$. Figure 6A represents the birth-death process and Figure 6B the reversible isomerization process. We see that the reversible isomerization process contains a significantly larger parametric region that corresponds to $\mathcal {P_{E}} > 0.80$, and that the probability in this region changes slowly (*i.e.*, plateau-like contour). Only two over-perturbing initial reaction rates (*i.e.,* orange squares above the green dotted line corresponding to $\mathcal {P_{E}} + \epsilon _{\mathcal {P_{E}}} = 0.85$) were generated for the birth-death process while eleven such rates were generated for the reversible isomerization process. Intermediate reaction rates (white squares) of these eleven samples are close together due to the slowly changing probability in their vicinity. Lastly we note that none of the data in Figure 6B left the original parameter ranges stated in the model description. This confirms that even for simple systems such as birth-death process and reversible isomerization process, it is nontrivial to predict parameter ranges that form a convex bound.

**Table 3:** **Results of SParSE applied to the reversible isomerization process**

$\boldsymbol{\mathcal {P_{E}}}$	$\epsilon _{\boldsymbol{\mathcal {P_{E}}}}$	**SParSE samples**	**Interpolations**	**Failures**	**Successful**
					**URS**
0.40	0.01	269 (50)	26 [4 9 14 3]	0	2
	0.05	181 (48)	18 [12 17 1 0]	0	6
	0.10	148 (44)	13 [17 13 0 0]	0	8
0.60	0.01	313 (58)	28 [2 6 16 6]	0	4
	0.05	198 (51)	18 [12 14 4 0]	0	8
	0.10	156 (47)	13 [17 13 0 0]	0	11
0.80	0.01	290 (75)	27 [3 17 5 5]	0	1
	0.05	201 (67)	12 [18 11 1 0]	0	6
	0.10	165 (61)	4 [26 4 0 0]	0	18

The column identities match those of Table 1. *N* = 5 × 10^4^.

Next we choose a high probability target of $\mathcal {P_{E}} = 0.95$ and $\epsilon _{\mathcal {P_{E}}} = 0.005$ with *N*=10^5^. Table 4 summarizes the result. We see that one of 30 samples failed to converge. SParSE was not able to find ***k***^∗^ for $\boldsymbol {k}^{(0)}_{27} = \left [0.205 \ 0.414\right ]$ after 3 rounds of exponential interpolations. The qualitative explanation for the failure is the same as with the birth-death process for $\mathcal {P_{E}} = 0.60$ and $\epsilon _{\mathcal {P_{E}}} = 0.01$, which is discussed in Appendix C of Additional file 1.

**Table 4:** **Results of SParSE applied to the reversible isomerization process**

$\boldsymbol{\mathcal {P_{E}}}$	$\epsilon _{\boldsymbol{\mathcal {P_{E}}}}$	**SParSE samples**	**Interpolations**	**Failures**	**Successful**
					**URS**
0.95	0.005	302 (109)	10 [20, 3, 4, 3]	1	2

The column identities match those of Table 1. *N*=1×10^5^.

It is worth pointing out that the number of successful parameter sets generated with SSA-URS varies widely from simulation to simulation. The expected number, however, is the volume of the solution hyperplane (which changes with different values of $\epsilon _{\mathcal {P_{E}}}\!$) divided by the total volume, multiplied by the total number of reaction rate samples generated with URS. Here the prescribed parameter ranges are used to compute both volumes (*e.g.*, 0.1≤*k*_1_≤0.3 and 0.3≤*k*_2_≤1.0 for the reversible isomerization process). Since the acceptable solution volume increases with larger $\epsilon _{\mathcal {P_{E}}}$, the number of uniform random samples that reside in the solution hyperplane should increase as well. Similarly the expected number of intermediate reaction rates used by SParSE to reach the solution hyperplane decreases because the need for fine-tuning, *i.e.*, exponential interpolation, declines with larger $\epsilon _{\mathcal {P_{E}}}$. This trend is confirmed by the simulation results for all three examples presented in this paper (columns 4 and 6 of Tables 1, 3, and 5).

**Table 5:** **Results of SParSE applied to the SIRS model**

$\boldsymbol{\mathcal {P_{E}}}$	$\epsilon _{\boldsymbol{\mathcal {P_{E}}}}$	**SParSE samples**	**Interpolations**	**Failures**	**Successful**
					**URS**
0.4	0.01	467 (162)	26 [4, 15, 8, 3]	0	5
	0.05	282 (149)	8 [22, 6, 1, 1]	0	6
	0.10	246 (142)	4 [26, 4, 0, 0]	0	14
0.6	0.01	318 (63)	28 [ 2, 8, 18, 2]	0	3
	0.05	206 (59)	20 [10, 19, 0, 1]	0	8
	0.10	166 (57)	10 [20, 9, 0, 1]	0	8
0.8	0.01	328 (113)	8 [22, 4, 3, 1]	0	4
	0.05	224 (90)	1 [29, 0, 0, 1]	0	26
	0.10	177 (73)	0 [30, 0, 0, 0]	0	41

The column identities match those of Table 1. *N* = 5 × 10^4^.

### Simple SIRS disease dynamics

**Figure 7:**
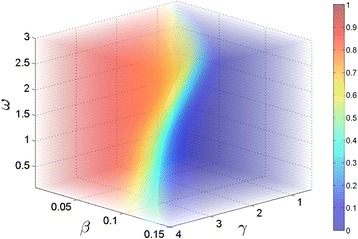
**Probability volume (70×70×70 grid) for the SIRS model after applying Matlab’s interp3 function to the 30×30×30 data simulated with SSA.** Color of each voxel represents $\mathcal {P_{E}}$ given its *β*, *γ*, and *ω* values according to the color bar given on the right.

The final example is Susceptible-Infectious-Recovered-Susceptible (SIRS) disease transmission model, which consists of the following three reactions:

$$\begin{aligned} S + I & \stackrel{\beta}{\rightarrow} 2I,& 0.005 \leq & \beta \leq 0.150 \\ I & \stackrel{\gamma}{\rightarrow} R, & 0.50 \leq & \gamma \leq 4.0\\ R & \stackrel{\omega}{\rightarrow} S, & 0.10 \leq & \omega \leq 3.0 \end{aligned} $$

with **x**_0_=[100 1 0], where **x**=[*SIR*]. This model describes a homogenous, fixed population setting where members of *S* become infected by members of *I*, who recover from the infection at rate *γ*. Once recovered, members of *R* have immunity against the infection. However, the immunity wanes at rate *ω*, and this transition from recovered to susceptible compartment replenishes the population of *S*. The target event for this system is set to the population of *I* reaching 50 before *t*=30. Unlike the first two examples, there is no closed-form analytical solution for this model. In order to construct the probability voxel for the specified parameter ranges, we divided each parameter region into 30 uniformly-spaced grids and computed each combination with the SSA, where each of 27,000(30^3^) ensembles was simulated with *N*=10^5^. We then further refined the resolution of the probability volume to a 70×70×70 grid using **interp3** function in Matlab, which applied linear interpolation to the 3-dimensional mesh data from SSA simulations. Figure 7 displays the final solution volume, where the color of each point represents the target event probability according to the color bar on the right of the figure.

As with the previous examples, we tested SParSE on all possible combinations of $\mathcal {P_{E}} \in \{0.40, \ 0.60, \ 0.80\}$ and $\epsilon _{\mathcal {P_{E}}} \in \{0.01, \ 0.05, \ 0.10\}$ and measured the same quantities as in Table 1. Table 5 summarizes the results. We see that SParSE achieved 100% success rate for all scenarios tested. However, statistics on column 1 demonstrates that the total number of estimates computed for any SIRS scenario is greater than the one for the first two examples with the same target probability and error tolerance. SIRS ensembles required up to 198 more samples, except for $\mathcal {P_{E}} =60$ and $\epsilon _{\mathcal {P_{E}}}=0.01$, which required one fewer sample than the birth-death process. If we ignore the intermediate event computations, the number of samples required by all three examples are comparable to each other (mean difference of 17.7 samples). In addition, quantities in column 4 of Tables 1, 3 and 5 indicate that SParSE required fewer interpolations on the SIRS model than it did on the other two examples. These results imply that the multilevel cross-entropy method applied to the SIRS model made conservative moves to reach the solution hyperplane; the algorithm required many intermediate event computations to approach the vicinity of $\mathcal {P_{E}}$ but fewer fine-tuning steps (*i.e.*, exponential interpolations). Although the same ***ρ***(*δ*) values were used for all three examples, we see that its effect differs depending on the underlying system.

Two expected trends emerge from Table 5; the total number of SParSE samples and the total number of exponential interpolations required to reach the solution hyperplane decrease with increasing $\epsilon _{\mathcal {P_{E}}}$. Although numbers in columns 3 and 4 differ among Tables 1, 3, and 5, qualitative algorithmic behavior as a function of $\epsilon _{\mathcal {P_{E}}}$ remain the same for all three examples. As for its performance, SParSE outperformed SSA-URS (by a factor of 1.15 to 10) on all scenarios except one. For $\mathcal {P_{E}} =.80$ and $\epsilon _{\mathcal {P_{E}}} = 0.10$, SSA with URS yielded 41 successful sets, while SParSE yielded 30. We note that the maximum number of successful sets for SParSE cannot exceed the number of initial parameter sets, which is 30 for all examples presented in this paper. Also, the parameter ranges we chose for the SIRS model result in an uneven distribution of the target probability. From Figure 7, we see that a significant portion of the probability volume belongs to high (>0.8) or low (<0.2) probability region. Since the SSA-URS success probability is determined solely by the ratio between the volume of the solution hyperplane and the total volume defined by the specified parameter ranges, this particular scenario is biased to be more favorable toward SSA-URS. For general applications involving a target event, however, we cannot expect the solution hyperplane to lie within the user-specified parameter ranges, to which SSA-URS samples are confined. If this region does not contain the solution hyperplane, SSA-URS is unable to produce ***k***^∗^ regardless of the number of samples generated. The current implementation of SParSE, on the other hand, is highly likely to find the closest point (cross-entropy metric) in the solution hyperplane through multilevel cross-entropy method and exponential interpolation stages, both of which are not limited by the user-specified parameter ranges. In practical situations, it is likely that the user does not have enough systematic insight to identify a region that contains the solution hyperplane for a particular target event. We expect SParSE to be more efficient than SSA-URS by orders of magnitude in such aligned, as the performance of SParSE is much less sensitive to the dimensionality of the search space and the volume within $\epsilon _{\mathcal {P_{E}}}$ of the solution hyperplane than the performance of SSA-URS is.

**Figure 8:**
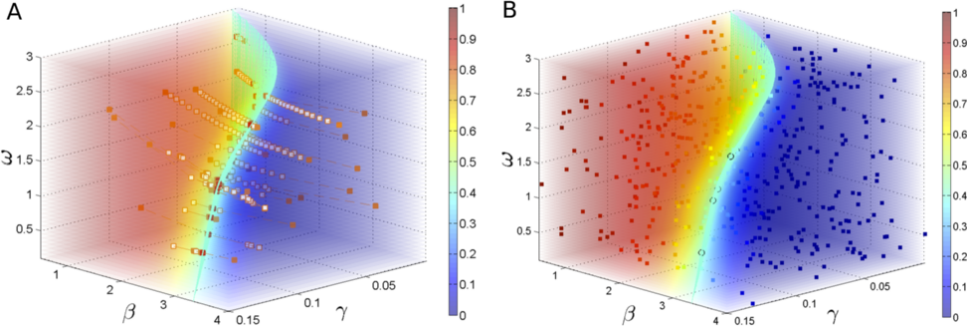
**SIRS ensemble result comparison between SParSE and SSA-URS for** $\boldsymbol {\boldsymbol{\mathcal {P_{E}}} = 0{.}40, \ \epsilon _{\boldsymbol{\mathcal {P_{E}}}} = 0{.}01}$ **, and** ***N=5×10*** ^***4***^ **.** Cyan-colored surface represents the solution hyperplane for the target event. **A** represents the result of SParSE applied to the SIRS model. Orange squares represent the initial reaction rates, white squares intermediate reaction rates, and red squares the final reaction rates on the solution hyperplane. Orange dashed lines connect any two subsequent reaction rates originated from the same initial reaction rates. **B** represents the result of SSA-URS applied to the SIRS model. Color of each rectangle represents the target event probability from the represented parameter combination according to the color bar given on the right. Out of 467 samples, only 5 lie on the solution hyperplane. These 5 successful parameter sets are encircled in black. Due to the 3-dimensional nature of this figure, there is no single angle where both the solution hyperplane and the 5 sets are easily visible.

We picked one scenario, $\mathcal {P_{E}} = 0.40$ and $\epsilon _{\mathcal {P_{E}}} = 0.05$, for visual comparison between SParSE and SSA-URS outcomes outcomes (animated illustration of SParSE simulations for this scenario is provided in Additional file 3). Figure 8 display the ensemble result for each method. The solution hyperplane for $\mathcal {P_{E}} = 0.40$ is represented by a cyan-colored surface, which was obtained by applying **isosurface** function in Matlab to the probability volume. We have omitted displaying the region corresponding to $\mathcal {P_{E}} \pm \epsilon _{\mathcal {P_{E}}}$ for clear visualization of data. We see that the volume of the solution hyperplane for this particular scenario is small relative to the volume of the entire voxel. Thus we expect poor performance from SSA-URS, which is confirmed by statistics in Table 5. SSA-URS generated only 5 successful parameter combinations out of 467 samples, while SParSE generated 30. Since one point in the solution hyperplane corresponds to one set of initial reaction rates in SParSE, this indicates 100% convergence. The number of data in Figure 8A and 8B are 305 and 467, respectively. Figure B contains 162 more data to compensate for the total number of intermediate event computations required by SParSE. Despite having fewer data, SParSE produced not only 6 times the number of ***k***^∗^ but also data that are closely scattered around the solution hyperplane. The latter fact offers couple advantages. First, having good resolution near $\mathcal {P_{E}} \pm \epsilon _{\mathcal {P_{E}}}$ enables more accurate mapping of the solution hyperplane, as it is unknown or computationally infeasible to be computed even for moderately sized systems (*e.g.*, 5–20 parameters). In addition if we were to run another set of simulations on the identical scenario, we can generate initial reaction rates that are near the solution hyperplane using the past simulation results.

**Figure 9:**
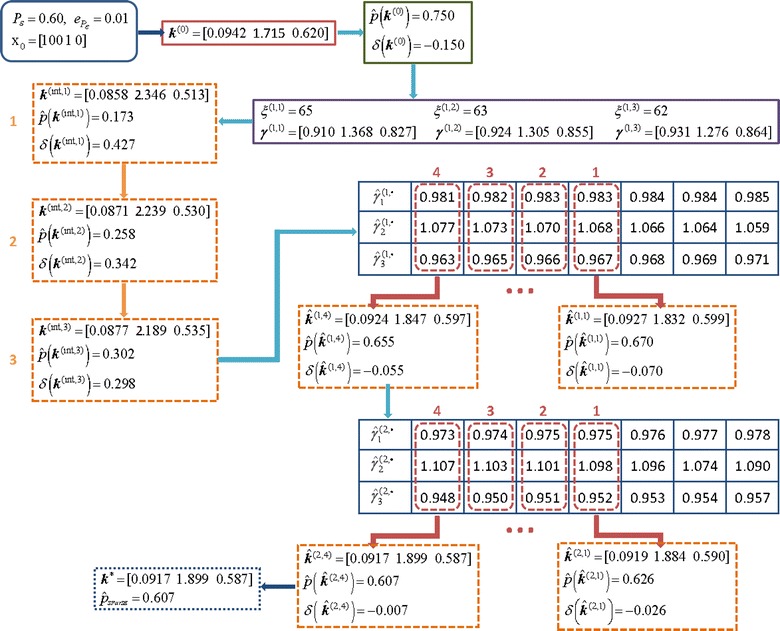
**Flow chart of SParSE simulation on the SIRS model with** $\boldsymbol{\mathcal {P_{E}}} = 0{.}60, \ \epsilon _{\boldsymbol{\mathcal {P_{E}}}} = 0{.}01, k^{(0)} = \left [0{.}0942 \; 1{.}7150 \; 0{.}6196\right ]$ **, and** ***N*** **=5×10** ^**4**^ **.** This particular simulation required three stages of multilevel cross-entropy method and two rounds of exponential interpolation. Arrows represent sequential steps taken by the algorithm.

**Figure 10:**
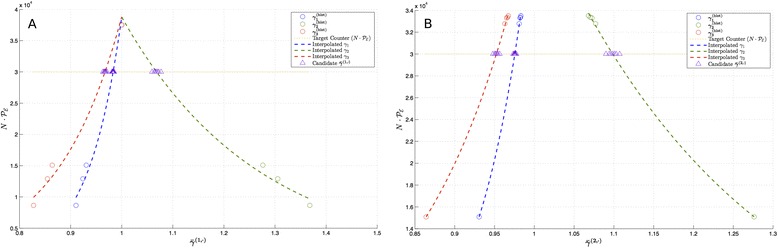
**Illustrations of first exponential interpolation (A) and second exponential interpolation (B) for the SIRS model with** $\boldsymbol{\boldsymbol{\mathcal {P_{E}}}} = 0{.}60$ **,** $\epsilon _{\boldsymbol{\boldsymbol{\mathcal {P_{E}}}}} = 0{.}01, k^{(0)} = \,[\!0{.}0942 \; 1{.}7150 \; 0{.}6196]$ **, and** ***N*** **=5×10** ^**4**^ **.** Yellow horizontal dotted line is the desired number of successful trajectories, *i.e.*, $N \times \mathcal {P_{E}} = 3 \times 10^{4}$. Blue, green, and red circles denote the past intermediate biasing parameters for *R*_1_, *R*_2_, and *R*_3_, respectively, normalized with respect to *k* ^(0)^. Blue, green, and red dashed lines are the interpolants constructed from the past intermediate basing parameters, and the purple triangles are candidate biasing parameters computed according to Step 3 in Subsection *Updating intermediate reaction rates*.

Lastly, we chose one of 30 initial reaction rates to illustrate a complete progression of SParSE on the SIRS model. Unlike the set of initial reaction rates chosen for the birth-death process ($\boldsymbol {k}^{(0)}_{14} = \,[\!1.2414 \; 0.02445]$ with $\mathcal {P_{E}} = 0.60$ and $\epsilon _{\mathcal {P_{E}}} = 0.01$) in Figure 1, which under-perturbs the system, the set of initial reaction rates chosen here, $\boldsymbol {k}^{(0)}_{26} = \,[\!0.0942 \; 1.7150 \; 0.6196]$, over-perturbs the system. Figure 9 displays the flow chart of SParSE simulations for this scenario, and Figure 10 illustrates the results from the first and second exponential interpolations, respectively. The interpolants for all three reactions exhibit a good fit with respect to the past biasing parameters, and the quality of fit improves in the second iteration with updated past estimates closer to the target probability. According to the flow chart and Figure 10A, SParSE entered the first iteration of exponential interpolation with four past estimates, three of which under-perturbed the system (from multilevel cross-entropy method). After exhausting the candidate biasing parameters from the first iteration, all of which produced estimates greater than 0.61, SParSE entered a second iteration of exponential interpolation. At this point, the top five closest estimates to $\mathcal {P_{E}} = 0.60$ all came from over-perturbing biasing parameter sets. SParSE then removed the most over-perturbing set and inserted the least under-perturbing set in attempt to improve the quality of the interpolant. Figure 10B reflects these modifications. The last candidate from the second interpolation produced an estimate within $\epsilon _{\mathcal {P_{E}}} = 0.01$, at which point the algorithm exited with the final reaction rates $(\boldsymbol {k}^{*}_{(26)} = \left [0.0917 \ 1.899 \ 0.587\right ])$. We note that the slope of the interpolants in Figure 10 are opposite from the ones in Figure 2. This is because inverse biasing technique is used for over-perturbing reaction rates, as described by Equation 12.

## Conclusions

In this paper, we presented SParSE–a novel stochastic parameter estimation algorithm for events. SParSE contains two main research contributions. First, it presents a novel modification of the multilevel cross-entropy method that (1) concurrently computes multiple intermediate events as well as their corresponding biasing parameters, and (2) handles over-perturbing initial reaction rates as well as under-perturbing ones. Second, it uses information from past simulations to automatically find a path to the parametric hyperplane corresponding to the target event with user-specified probability and absolute error tolerance.

By introducing a novel heuristic for handling reaction rates that over-perturb the system, SParSE can handle target events whose probability does not need to be rare with respect to the initial reaction rates ***k***^(0)^. If the user wishes to compute the probability of observing $\mathcal {P_{E}}$ with respect to ***k***^(0)^, however, it can be done by simply running the dwSSA with biasing parameters that are the ratio between the final reaction rates ***k***^∗^ from SParSE and ***k***^(0)^. No additional multilevel cross-entropy simulations are required by the dwSSA to determine biasing parameters since the final set of reaction rates computed by SParSE contains this information. For this reason, SParSE improves upon the dwSSA in that it can handle an additional type of rare event. The only class of rare events whose probability dwSSA can estimate is the one that is seldom reached by the system using the original reaction rates. SParSE, on the other hand, can also compute the probability of events that are reached too often with respect to the target probability using the original reaction rates. Average frequency of observing such target event with ***k***^(0)^ would be much higher than the desired frequency (*i.e.*, $(\mathcal {P_{E}} \pm \epsilon _{\mathcal {P_{E}}}) \times N$), and therefore the probability of observing  with success rate $\mathcal {P_{E}} \pm \epsilon _{\mathcal {P_{E}}}$ and reaction rates ***k***^(0)^ would be very small, yet its biasing parameters are uncomputable with the dwSSA, but are computable with SParSE.

It is important to note that the computational complexity of SParSE is *independent* of the number of parameters to be estimated. Like the dwSSA [17], SParSE utilizes information-theoretic concept of cross-entropy to concurrently compute biasing parameters for all reactions in the system. Moreover, SParSE avoids serial computation of biasing parameters for multiple intermediate events at any given stage of multilevel cross-entropy method by introducing a clever ordering of intermediate events and data management. Figures 4, 5 and 8 illustrate that SParSE not only is more efficient than SSA-URS in finding ***k***^∗^ but also gives a better resolution of the area near the solution hyperplane. This is because intermediate reaction rates computed by SParSE are guaranteed to be closer to ***k***^∗^ than ***k***^(0)^ is. Thus intermediate reaction rates near ***k***^∗^ can be used to improve the quality of interpolation in constructing the solution hyperplane. Another computational asset of SParSE is that it is highly parallelizable. In large scale application, multiple sets of initial reaction rates can be dispatched separately since each set finds its way to the solution hyperplane independently from each other. In smaller scale, SParSE estimate computation or an ensemble of multilevel cross-entropy method simulations also can be parallelized. In simulating examples presented in this paper, we have chosen the latter method; each set of *N* simulations was distributed among 8 cores using the Parallel Computing Toolbox™ in Matlab. Lastly, a single SParSE trajectory from the multilevel cross-entropy method without any biasing (*i.e.*,$\boldsymbol {\gamma } = \overrightarrow {\boldsymbol 1}$) generates the same number of uniform random numbers as the SSA does. The only difference is that SParSE requires additional data management for recording biasing parameter information (two floating point numbers for each reaction [17]), which is used in the next round of multilevel cross-entropy method. It is difficult to compare the exact computational cost between the two methods when SParSE utilizes $\boldsymbol {\gamma } \neq \overrightarrow {\boldsymbol 1}$; depending on the amount of bias applied per reaction, the number of random numbers generated per trajectory will differ between the two methods even if the same reaction rates were used. For the exponential interpolation stage in SParSE, SSA is used to compute $\hat {p}_{\textit {SParSE}}$, thus the computational cost of SParSE and SSA trajectory are identical for a given set of reaction rates.

One of the inputs required by SParSE is a range of values each parameter can take. There is no theoretical limit on the parameter range SParSE can manage; however, it is required for the following practical reasons. First, the volume of the solution hyperplane could be infinite if we do not confine parameter ranges. For the reversible isomerization process presented in the previous section, all solution hyperplanes from the 9 standard test scenarios are defined by the ratio between the two reaction rate parameters; infinitely many pairs exist that keep this ratio conserved. In addition, a range is required to sample initial reaction rates. If a user wishes to use a distribution other than the uniform distribution to generate initial reaction rates, different statistics (mean, standard deviation, *etc.*) may be needed.

We remind our readers that although parameter ranges are used to constrain the position of initial reaction rates, the same ranges are *not* enforced on the final reaction rates on the solution hyperplane. The main reason for this is that there is no guarantee the solution hyperplane intersects with the volume defined by the user-specified parameter ranges. By not limiting the final reaction rates to reside within the user-specified region, SParSE is able to find a set of reaction rates that lie on the solution hyperplane that are close to the user-specified parameter ranges. For example, in Figure 3, white dashed lines represent parameter ranges specified prior to the simulation. We see that 3 of 30 initial sets reached the solution hyperplane but are outside this region. We also see that some intermediate reaction rates (white squares) escape the region but return to it by the time ***k***^∗^ (red square) is found. For most practical applications, we know neither the curvature of the solution hyperplane nor the existence of it within the prescribed parameter ranges. The parameter ranges for all examples in this paper were chosen such that all possible values in (0 1) are captured while the volume of a solution hyperplane for any particular $\mathcal {P_{E}}$ is well-defined within this region. Therefore we expect the computational gain from employing SParSE over SSA-URS to be much higher for an arbitrary problem where the user is unable to provide informative parameter ranges for the target event of interest and its desired probability.

Future work will focus on two main areas whose improvement will substantially benefit the algorithm. First, the multilevel cross-entropy method for SParSE can improve from employing an adaptive ***ρ***(*δ*) function, whose values for determining intermediate events would change as the simulation progresses. While SParSE proved to be computationally efficient for all three examples presented in this paper, their results demonstrated that the same ***ρ***(*δ*) function can produce qualitatively different behavior on how the system approaches the solution hyperplane. We can use past values of ***ρ***(*δ*) and its effect on $\hat {p}_{\textit {SParSE}}$ to estimate the speed of convergence toward the solution hyperplane. This can potentially reduce the number of multilevel cross-entropy method iterations, where reduction of each iteration saves 2×*N* simulations. The second area of future research will be on efficient sampling of initial reaction rates. Once SParSE finishes simulating first sets of ***k***^(0)^, positions of resulting ***k***^∗^ may be far away from each other and thus insufficient to construct an accurate picture of the solution hyperplane. Instead of randomly sampling the next set of initial reaction rates, we can utilize information from the prior ensemble of SParSE simulations to improve the positioning of the next set of ***k***^(0)^. For example, we can construct a rough interpolation (*e.g.*, linear interpolation) of the solution hyperplane using ***k***^∗^s from the first ensemble, and sample the next set from the estimated solution hyperplane, which could be constrained by the user-specified parameter ranges if necessary. A more sophisticated method would be required for high-dimensional systems or for target events with discontinuity in the solution hyperplane.

## Additional files

Additional file 1
**Appendix.** Appendix is composed of three parts: tables, pseudocode, and discussion of failure in convergence. Table I contains the definition of variables used in Methods Section, and Table II provides a list of input parameters for SParSE and its default value when applicable. Table III contains the definition of variables exclusively used in pseudocode. In the second part, SParSE pseudocode is provided in a format of five separate algorithms for easy of reading and reproducibility. Lastly, we discuss in detail the three failures in Results and discussion Section.

Additional file 2
**This file contains an animated illustration of SParSE simulations for Birth-death process with** $\boldsymbol{\boldsymbol{\mathcal {P_{E}}}} =\, 0.010$ **and** $\epsilon _{\boldsymbol{\boldsymbol{\mathcal {P_{E}}}}}= \, 0.001.$

Additional file 3
**This file contains an animated illustration of SParSE simulations for SIRS disease dynamics model with** $\boldsymbol{\boldsymbol{\mathcal {P_{E}}}} =\, 0.40$ **and** $\epsilon _{\boldsymbol{\boldsymbol{\mathcal {P_{E}}}}} =\, 0.01.$
