# Supplementary material for: Stochastic parameter search for events
Source: BMC Syst Biol. 2014 Nov 8;8:126. doi: 10.1186/s12918-014-0126-y (PMC4229623; doi:10.1186/s12918-014-0126-y)
Supplement: Additional file 1 — Appendix. Appendix is composed of three parts: tables, pseudocode, and discussion of failure in convergence. Table I contains the definition of variables used in Methods Section, and Table II provides a list of input parameters for SParSE and its default value when applicable. Table III contains the definition of variables exclusively used in pseudocode. In the second part, SParSE pseudocode is provided in a format of five separate algorithms for easy of reading and reproducibility. Lastly, we discuss in detail the three failures in Results and discussion Section. [file 12918_2014_126_MOESM1_ESM.pdf]

Appendices

A. REFERENCE TABLES

TABLE I: Table of definitions

Variable	Definition
$\mathcal{T}$	$\min\Big(t_{final}, \Big(\min_t \lvert I_{\{f(\mathbf{x}(t\lvert\boldsymbol{k}))\cap\mathcal{E}\}} = 1\Big)\Big)$
$N_{\mathcal{T}}$	total number of reactions fired in the interval $[0, \mathcal{T}]$
$\delta(\boldsymbol{k})$	distance from the target, $\mathcal{P}_{\mathcal{E}} - \hat{p}_{SParSE}(\boldsymbol{k})$
$\boldsymbol{\xi}$	intermediate events
$\boldsymbol{\rho}$	fractions used to compute intermediate events Eqs. (10, 11)
$\eta(\boldsymbol{k})$	$\sum_{i=1}^{N}\left[I_{\{f(\mathbf{x}_i(t\lvert\boldsymbol{k}))\cap\mathcal{E}\}}\right]$
$\hat{p}_{SParSE}(\boldsymbol{k})$	$\frac{1}{N}\ \eta(\boldsymbol{k})$

TABLE II: List of input parameters

Parameter	Default value	Description
$\mathcal{E}$	NA	target event
$f(\mathbf{x}(t))$	NA	event (distance) function
$\mathcal{P}_{\mathcal{E}}$	NA	target probability for $\mathcal{E}$
$\epsilon_{\mathcal{P}_{\mathcal{E}}}$	0.05	absolute error tolerance
N	$5 \cdot 10^4$	number of simulations in computing $\hat{p}_{SParSE}(\boldsymbol{k})$
$\mathcal{L}$	10	maximum number of multilevel CE method per $\boldsymbol{k}^{(0)}$
$\mathcal{I}$	3	maximum number of exponential interpolation per $\boldsymbol{k}^{(0)}$

TABLE III: Table of definitions for variables used in **Algorithms 1-5**

Variable	Definition
M	number of reactions in the system
$\boldsymbol{\gamma}^{\text{hist}}$	matrix of past intermediate biasing parameter values normalized with respect to $\boldsymbol{k}^{(0)}$. $\gamma^{\text{hist}}_{(i,j)}$ denotes i^{th} past intermediate biasing parameter for R_j.
$\boldsymbol{\eta}^{\text{hist}}$	vector of past η values computed. η^{hist}_i denotes i^{th} past η value.
$\boldsymbol{\gamma}^{\text{num}}$	matrix whose entries are used to accumulate the numerator in Eq. (13) matrix of size $q \times M$, where q is the number of intermediate events $\gamma^{\text{num}}_{(i,j)}$ denotes values of the matrix at row i and column j.
$\boldsymbol{\gamma}^{\text{denom}}$	matrix whose entries are used to accumulate the denominator in Eq. (13) matrix of size $q \times M$, where q is the number of intermediate events $\gamma^{\text{denom}}_{(i,j)}$ denotes values of the matrix at row i and column j.
$\bar{\boldsymbol{k}}$	matrix of candidate reaction rates from executing Steps 1-3 in Exponential Interpolation of Biasing Parameters matrix of size $r \times M$, where r is the length of the candidate reaction rates $\bar{k}^{(l,s)}_j$ denotes s^{th} candidate reaction rate for R_j in l^{th} interpolation.
$\bar{\boldsymbol{\gamma}}$	matrix of candidate biasing parameter rates from fitting exponential interpolant and executing Step 1. matrix of size $r \times M$, where r is the length of the candidate biasing parameters $\bar{\gamma}^{(l,s)}_j$ denotes s^{th} candidate biasing parameter for R_j in l^{th} interpolation.

B. SPARSE PSEUDOCODE

In the below pseudocode, $\boldsymbol{\nu}_j$ and t_f represent the state change vector for reaction j and the simulation end time, respectively. The maximum number of multilevel cross-entropy method for any given set of initial reaction rates is denoted by $\mathcal{L}$, and exceeding $\mathcal{L}$ is considered a failure in convergence. Similarly, the maximum number of exponential interpolation is denoted by $\mathcal{I}$. The computational complexity of Algorithms 2-4 is identical to that of dwSSA.

Algorithm 1. SParSE Driver

Input: $\boldsymbol{k}^{(0)},\ \mathcal{L},\ \mathcal{E},\ N,\ \mathcal{P}_{\mathcal{E}},\ \epsilon_{\mathcal{P}_{\mathcal{E}}}$

  $l \leftarrow 0$
2: $\phi_{\text{type}} \leftarrow$ Eq. (8)
  $\boldsymbol{k}^{(\text{cur})} \leftarrow \boldsymbol{k}^{(0)}, \quad \boldsymbol{\gamma}^{(l)} \leftarrow$ matrix of size $1 \times M$
4: $\boldsymbol{\gamma}^{\text{hist}},\ \boldsymbol{\eta}^{\text{hist}} \leftarrow \emptyset$
  doInterpolate, isOverPerturbed $\leftarrow$ **false**
6: **while** $l < \mathcal{L}$ **do**
  for $i = 1$ to number of rows in $\boldsymbol{\gamma}^{(l)}$ **do**
8: $\boldsymbol{\gamma}^{(\text{l,i})} \leftarrow i^{th}$ row of $\boldsymbol{\gamma}^{(l,\cdot)}, \quad \boldsymbol{k}^{(\text{int},i)} \leftarrow$ Eq. (12)
  $\boldsymbol{\xi},\ \eta\left(\boldsymbol{k}^{(\text{int},i)}\right) \leftarrow$ **Algorithm 2**
10: $\hat{p}_{SParSE}\left(\boldsymbol{k}^{(\text{int},i)}\right) = \frac{1}{N} \cdot \eta\left(\boldsymbol{k}^{(\text{int},i)}\right), \quad \delta\left(\boldsymbol{k}^{(\text{int},i)}\right) = \mathcal{P}_{\mathcal{E}} - \hat{p}_{SParSE}\left(\boldsymbol{k}^{(\text{int},i)}\right)$
  if $|\delta\left(\boldsymbol{k}^{(\text{int},i)}\right)| < \epsilon_{\mathcal{P}_{\mathcal{E}}}$ **then**
12: $\boldsymbol{k}^{*} \leftarrow \boldsymbol{k}^{(\text{int},i)}$
  go to **step** (90)
14: **end if**
  $\boldsymbol{\gamma}^{\text{hist}} |\ \gamma^{\text{hist}}_{(\text{end}+1,j)} = k_j^{(\text{int},i)}/k_j^{(0)},\ j = \{1, \cdots, M\}$
16: $\boldsymbol{\eta}^{\text{hist}} |\ \eta^{\text{hist}}_{\text{end}+1} = \eta\left(\boldsymbol{k}^{(\text{int},i)}\right)$
  if isOverPerturbed == **false then**
18: **if** sgn $\left(\delta\left(\boldsymbol{k}^{(\text{int},i)}\right)\right) \neq \phi_{\text{type}}$ **then** {reached over perturbed state}
  if $l ==$ number of rows in $\boldsymbol{\gamma}^{(l)}$ **then**
20: **if** $\left(\forall\ \eta \in \boldsymbol{\eta}^{\text{hist}},\ \eta > \mathcal{P}_{\mathcal{E}} \cdot N\right)$ **then**
  isOverPerturbed $\leftarrow$ **true**
22: **else**
  break out of **while** loop in **step** (6)
24: **end if**
  else
26: doInterpolate $\leftarrow$ **true**, continue **for** loop in **step** (7)
  end if
28: **end if**
  if doInterpolate == **true then**
30: break out of **while** loop in **step** (6)
  else

$\boldsymbol{k}^{(\mathrm{cur})} \leftarrow \boldsymbol{k}^{(\mathrm{int},i)}$

break out of **for** loop in **step** (7)

end if

end if

if isOverPerturbed == **true then**

if $\mathrm{sgn}\left(\delta\left(\boldsymbol{k}^{(\mathrm{int},i)}\right)\right) == \phi_{\mathrm{type}}$ **then** {reached under perturbed state}

if l == number of rows in $\boldsymbol{\gamma}^{(l,\cdot)}$ **then**

break out of **while** loop in **step** (6)

else

doInterpolate ← **true**,   continue **for** loop in **step** (7)

end if

else

if doInterpolate == **true then**

break out of **while** loop in **step** (6)

else

$\boldsymbol{k}^{(\mathrm{cur})} \leftarrow \boldsymbol{k}^{(\mathrm{int},i)}$, $\boldsymbol{\xi} \leftarrow$ **Algorithm 3**

break out of **for** loop in **step** (7)

end if

end if

end if

end for

$\boldsymbol{\gamma}^{\mathrm{num}},\ \boldsymbol{\gamma}^{\mathrm{denom}} \leftarrow$ **Algorithm 4** with $\boldsymbol{k}^{(\mathrm{cur})}$

$l \leftarrow l + 1$

if isOverPerturbed == **false then**

$\boldsymbol{\gamma}^{(l)} | \gamma^{(l)}_{(i,j)} = \gamma^{\mathrm{num}}_{(i,j)} / \gamma^{\mathrm{denom}}_{(i,j)}$

else {inverse biasing}

$\boldsymbol{\gamma}^{(l)} | \gamma^{(l)}_{(i,j)} = \gamma^{\mathrm{denom}}_{(i,j)} / \gamma^{\mathrm{num}}_{(i,j)}$

end if

end while

retry ← 1 {used to count the number of interpolation stage}

$\bar{\boldsymbol{k}}^{(\mathrm{retry},\cdot)}$, $\mathrm{ind}_{\bar{\boldsymbol{k}}} \leftarrow$ **Algorithm 5**

while retry $\leq \mathcal{I}$ **do**

64: $\boldsymbol{k}^{(\text{cur})} \leftarrow \bar{\boldsymbol{k}}^{(\text{retry},\text{ind}_{\bar{\boldsymbol{k}}})}$

$\boldsymbol{\xi},\ \eta\left(\boldsymbol{k}^{(\text{cur})}\right) \leftarrow$ **Algorithm 2**

66: $\hat{p}_{SParSE}\left(\boldsymbol{k}^{(\text{cur})}\right) = \frac{1}{N} \cdot \eta\left(\boldsymbol{k}^{(\text{cur})}\right), \quad \delta\left(\boldsymbol{k}^{(\text{cur})}\right) = \mathcal{P}_{\mathcal{E}} - \hat{p}_{SParSE}\left(\boldsymbol{k}^{(\text{cur})}\right)$

if $\text{ind}_{\bar{\boldsymbol{k}}}$ was visited twice before **then**

68: **if** weighted average was tried **then**

Failure in convergence. Exit **Algorithm 1**.

70: **else**

$\boldsymbol{k}^{(\text{cur})} \leftarrow$ weighted average between $\bar{\boldsymbol{k}}^{(\text{retry},\text{ind}_{\bar{\boldsymbol{k}}})}$ and the previous $\boldsymbol{k}^{(\text{cur})}$

72: **end if**

else

74: **if** $|\delta\left(\boldsymbol{k}^{(\text{cur})}\right)| < \epsilon_{\mathcal{P}_{\mathcal{E}}}$ **then**

$\boldsymbol{k}^* \leftarrow \boldsymbol{k}^{(\text{cur})}$

76: go to **step** (90)

end if

78: $\boldsymbol{\gamma}^{\text{hist}} |\ \gamma^{\text{hist}}_{(\text{end}+1,j)} = k_j^{(\text{cur})}/k_j^{(0)},\ j = \{1, \cdots, M\}$

$\boldsymbol{\eta}^{\text{hist}} |\ \eta^{\text{hist}}_{\text{end}+1} = \eta\left(\boldsymbol{k}^{(\text{cur})}\right)$

80: **if** $\delta\left(\boldsymbol{k}^{(\text{cur})}\right) < 0$ **and** $\text{ind}_\gamma > 1$ **then**

$\text{ind}_{\bar{\boldsymbol{k}}} \leftarrow \text{ind}_{\bar{\boldsymbol{k}}} - 1$

82: **else if** $\delta\left(\text{ind}_{\bar{\boldsymbol{k}}}\right) > 0$ **and** $\text{ind}_\gamma <$ number of rows in $\bar{\boldsymbol{k}}^{(\text{retry},\cdot)}$ **then**

$\text{ind}_{\bar{\boldsymbol{k}}} \leftarrow \text{ind}_{\bar{\boldsymbol{k}}} + 1$

84: **else**

retry $\leftarrow$ retry+1

86: $\bar{\boldsymbol{k}}^{(\text{retry},\cdot)},\ \text{ind}_{\bar{\boldsymbol{k}}} \leftarrow$ **Algorithm 5**

end if

88: **end if**

end while

90: **return** $\boldsymbol{k}^*$

Algorithm 2. Intermediate Event with Under Perturbation.

Input: $\boldsymbol{k},\ \mathcal{E},\ N,\ f(\mathbf{x}(t))$

$\mathbf{v}_{\boldsymbol{k}},\ \boldsymbol{I}_\xi \leftarrow \overrightarrow{\mathbf{0}}$ {zero vector of size N}

2: **for** $i = 1$ to N **do**

$t \leftarrow 0, \quad \mathbf{x} \leftarrow \mathbf{x}_0$

evaluate all $a_j(\mathbf{x}),\ j = \{1, \cdots, M\}$; calculate $a_0(\mathbf{x})$

$\mathbf{v}_{\boldsymbol{k}}[i] \leftarrow f\left(\mathbf{x}_0\right)$

while $t \le t_f$ **do**

if $f(\mathbf{x}) == \mathcal{E}$ **then**

$\boldsymbol{I}_{\xi}[i] \leftarrow 1$

break out of the while loop

end if

generate two unit-interval uniform random numbers r_1 and r_2

$\tau \leftarrow {a_0}^{-1}(\mathbf{x}) \ln(1/r_1)$

$j' \leftarrow$ smallest integer satisfying $\sum_{j=1}^{j'} a_j(\mathbf{x}) \ge r_2 a_0(\mathbf{x})$

$t \leftarrow t + \tau, \quad \mathbf{x} \leftarrow \mathbf{x} + \boldsymbol{\nu}_{j'}$

update all $a_j(\mathbf{x}),\ j = \{1, \cdots, M\}$; recalculate $a_0(\mathbf{x})$

if $\phi_{\text{type}} == 1$ **then**

$\mathbf{v}_{\boldsymbol{k}}[i] \leftarrow \max\left(\mathbf{v}_{\boldsymbol{k}}[i], f\left(\mathbf{x}\right)\right)$

else

$\mathbf{v}_{\boldsymbol{k}}[i] \leftarrow \min\left(\mathbf{v}_{\boldsymbol{k}}[i], f\left(\mathbf{x}\right)\right)$

end if

end while

end for

$\eta\left(\boldsymbol{k}\right) \leftarrow \sum_{i=1}^{N} \boldsymbol{I}_{\xi}[i], \quad \boldsymbol{\rho}(\delta) \leftarrow$ Eq. (10)

if $\phi_{\text{type}} == 1$ **then**

Sort $\mathbf{v}_{\boldsymbol{k}}$ in descending order

else

Sort $\mathbf{v}_{\boldsymbol{k}}$ in ascending order

end if

$\boldsymbol{\xi} \leftarrow$ unique elements of $\mathbf{v}_{\boldsymbol{k}}$ at indices $\lceil \boldsymbol{\rho}(\delta) \times N \rceil$

return $\boldsymbol{\xi}$ and $\eta\left(\boldsymbol{k}\right)$

Algorithm 3. Intermediate Event with Over Perturbation.

Input: $\boldsymbol{k}$, N

$\mathbf{v}_{\boldsymbol{k}} \leftarrow \overrightarrow{\mathbf{0}}$ {zero vector of size N}

for $i = 1$ to N **do**

  $t \leftarrow 0, \quad \mathbf{x} \leftarrow \mathbf{x}_0$

  evaluate all $a_j(\mathbf{x}),\ j = \{1, \cdots, M\}$; calculate $a_0(\mathbf{x})$

  $\mathbf{v}_{\boldsymbol{k}}[i] \leftarrow f(\mathbf{x}_0)$

  while $t \leq t_f$ **do**

    generate two unit-interval uniform random numbers r_1 and r_2

    $\tau \leftarrow {a_0}^{-1}(\mathbf{x}) \ln(1/r_1)$

    $j' \leftarrow$ smallest integer satisfying $\sum_{j=1}^{j'} a_j(\mathbf{x}) \geq r_2 a_0(\mathbf{x})$

    $t \leftarrow t + \tau, \quad \mathbf{x} \leftarrow \mathbf{x} + \boldsymbol{\nu}_{j'}$

    update all $a_j(\mathbf{x}),\ j = (1, \cdots, M)$; recalculate $a_0(\mathbf{x})$

    if $\phi_{\text{type}} == 1$ **then**

      $\mathbf{v}_{\boldsymbol{k}}[i] \leftarrow \max(\mathbf{v}_{\boldsymbol{k}}[i], f(\mathbf{x}))$

    else

      $\mathbf{v}_{\boldsymbol{k}}[i] \leftarrow \min(\mathbf{v}_{\boldsymbol{k}}[i], f(\mathbf{x}))$

    end if

  end while

end for

$\boldsymbol{\rho}(\delta) \leftarrow$ Eq. (11)

if $\phi_{\text{type}} == 1$ **then**

  Sort $\mathbf{v}_{\boldsymbol{k}}$ in descending order

else

  Sort $\mathbf{v}_{\boldsymbol{k}}$ in ascending order

end if

$\boldsymbol{\xi} \leftarrow$ unique elements of $\mathbf{v}_{\boldsymbol{k}}$ at indices $\lceil \boldsymbol{\rho}(\delta) \times N \rceil$

return $\boldsymbol{\xi}$

Algorithm 4. Biasing Parameter Computation.

Input: $\boldsymbol{k},\ \boldsymbol{\xi},\ N$

$q \leftarrow$ length of $\boldsymbol{\xi}$

$\boldsymbol{\gamma}^{\text{num}}, \boldsymbol{\gamma}^{\text{denom}} \leftarrow 0$ matrix of size $q \times M$

for $i = 1$ to N **do**

  $\boldsymbol{\gamma}^{\text{numi}}, \boldsymbol{\gamma}^{\text{denomi}} \leftarrow 0$ matrix of size $q \times M$

$\boldsymbol{n},\ \boldsymbol{\lambda} \leftarrow \overrightarrow{\mathbf{0}}$ {zero vector of size M}

6: $\mathrm{ind}_{IE} \leftarrow q$

$t \leftarrow 0, \quad \mathbf{x} \leftarrow \mathbf{x}_0$

8: evaluate all $a_j(\mathbf{x}),\ j = \{1, \cdots, M\}$; calculate $a_0(\mathbf{x})$

while $t \leq t_f$ **do**

10: **if** $f(\mathbf{x}) == \xi^{(\mathrm{ind}_{IE})}$ **then**

$\gamma^{\mathrm{numi}}_{(\mathrm{ind}_{IE},j)} \leftarrow n_j, \quad \gamma^{\mathrm{denomi}}_{(\mathrm{ind}_{IE},j)} \leftarrow \lambda_j, \quad j = \{1, \cdots, M\}$

12: **if** $\mathrm{ind}_{IE} == 1$ **then**

break out of **while** loop in **step** (9)

14: **else**

$\mathrm{ind}_{IE} = \mathrm{ind}_{IE} - 1$

16: **end if**

end if

18: generate two unit-interval uniform random numbers r_1 and r_2

$\tau \leftarrow {a_0}^{-1}(\mathbf{x}) \ln(1/r_1)$

20: $j' \leftarrow$ smallest integer satisfying $\sum_{j=1}^{j'} a_j(\mathbf{x}) \geq r_2 a_0(\mathbf{x})$

$t \leftarrow t + \tau, \quad \mathbf{x} \leftarrow \mathbf{x} + \boldsymbol{\nu}_{j'}$

22: update all $a_j(\mathbf{x}),\ j = (1, \cdots, M)$; recalculate $a_0(\mathbf{x})$

$n_{j'} \leftarrow n_{j'} + 1, \quad \lambda_j \leftarrow \lambda_j + a_j(\mathbf{x}) \cdot \tau, \quad j = (1, \cdots, M)$

24: **end while**

$\gamma^{\mathrm{num}}_{(i,j)} \leftarrow \gamma^{\mathrm{num}}_{(i,j)} + \gamma^{\mathrm{numi}}_{(i,j)}, \quad i = \{1, \cdots, q\},\ j = \{1, \cdots, M\}$

26: $\gamma^{\mathrm{denom}}_{(i,j)} \leftarrow \gamma^{\mathrm{denom}}_{(i,j)} + \gamma^{\mathrm{denomi}}_{(i,j)}, \quad i = \{1, \cdots, q\},\ j = \{1, \cdots, M\}$

end for

28: **return** $\boldsymbol{\gamma}^{\mathrm{num}}, \boldsymbol{\gamma}^{\mathrm{denom}}$

Algorithm 5. Exponential Interpolation of Biasing Parameters.

Input: $\mathcal{P}_{\mathcal{E}},\ \epsilon_{\mathcal{P}_{\mathcal{E}}},\ \boldsymbol{k}^{(0)},\ \boldsymbol{\gamma}^{\mathrm{hist}},\ \boldsymbol{\eta}^{\mathrm{hist}},\ N$

1: $\boldsymbol{\eta}^{\mathrm{interp}} \leftarrow ([-2\ \ -1\ \ -0.5\ 0\ 0.5\ 1\ 2] \cdot \epsilon_{\mathcal{P}_{\mathcal{E}}} + \mathcal{P}_{\mathcal{E}}) \times N$

2: [Optional] keep at most 5 elements in $\boldsymbol{\eta}^{\mathrm{hist}}$ closest to $\mathcal{P}_{\mathcal{E}} \cdot N$ and the corresponding $\boldsymbol{\gamma}^{\mathrm{hist}}$ with at least one element of $\boldsymbol{\eta}^{\mathrm{hist}} < \mathcal{P}_{\mathcal{E}} \cdot N$ and one element $> \mathcal{P}_{\mathcal{E}} \cdot N$

3: **for all** $j \in 1, \cdots, M$ **do**

4: solve for a and b in $\boldsymbol{y} = a\boldsymbol{x} + b$, where $\boldsymbol{x} \leftarrow \boldsymbol{\gamma}^{\mathrm{hist}}_{(\cdot,j)}$ and $\boldsymbol{y} \leftarrow \log\left(\boldsymbol{\eta}^{\mathrm{hist}}\right)$

$\bar{\gamma}_j^{(\cdot,s)} \leftarrow \left(\log\left(\eta_s^{\text{interp}}\right) - b\right)/a,\ s = \{1, \cdots, 7\}$

apply **Steps** 2-3 in Exponential Interpolation of Biasing Parameters and compute $\bar{k}_j^{(\cdot,s)}$

end for

remove duplicate sets in $\bar{\boldsymbol{k}}^{(\cdot,s)}$ if applicable

$\text{ind}_{\bar{\boldsymbol{k}}} \leftarrow$ index where $\eta^{\text{hist}}_{\text{ind}_{\bar{\boldsymbol{k}}}} == \mathcal{P}_{\mathcal{E}} \cdot N$

return $\bar{\boldsymbol{k}}$, $\text{ind}_{\bar{\boldsymbol{k}}}$

C. FAILURES IN CONVERGENCE

Results and Discussion section contains a total of 330 test cases of SParSE among three example systems provided: birth-death process, reversible isomerization process, and SIRS disease transmission model. Out of the 330 cases, we observed three failures in convergence: two in birth-death process and one in reversible isomerization process. For birth-death process with $\mathcal{P}_{\mathcal{E}} = 0.60$ and $\epsilon_{\mathcal{P}_{\mathcal{E}}} = 0.01$, two samples, $\boldsymbol{k}_3^{(0)} = [1.606\ 0.0140]$ and $\boldsymbol{k}_{27}^{(0)} = [1.684\ 0.0148]$ (subscript representing the index of initial reaction rates), failed to converge after three rounds of exponential interpolations. One failure in reversible isomerization is caused by $\boldsymbol{k}_{27}^{(0)} = [0.205\ 0.414]$ with $\mathcal{P}_{\mathcal{E}} = 0.95$ and $\epsilon_{\mathcal{P}_{\mathcal{E}}} = 0.005$. We note that the following discussion involves variables and procedures from **Methods** section.

For all three cases, the failure to converge was a result of poor agreement between the past simulation data and the corresponding interpolants. Figure C.1 displays the exponential interpolants from the first iteration for $\boldsymbol{k}_3^{(0)}$ and $\boldsymbol{k}_{27}^{(0)}$ from birth-death process. Plots of successive interpolations and of reversible isomerization exhibit similar qualitative fit and are omitted from display. We see from Figure C.1 that an interpolant cannot pass through the past data without changing its inflection, of which an exponential function is incapable. Consequently all 7 candidate biasing parameters underwent Step 3 to satisfy **Assumption 1** and yielded a single set after removing duplicates. At this point, the final set of candidate biasing parameters is the same as $\boldsymbol{k}^{(v)}$, and the resulting SParSE estimate supplies no additional information. Similar results occur in the next two subsequent interpolations. We note that for both failed cases, taking five past data sets closest to $\mathcal{P}_{\mathcal{E}}$ does not improve the quality of interpolation. This is because three sets of biasing parameters that underperturb the system (*i.e.*, below the yellow dotted line in Figure C.1) produce estimates that

are farther from $\mathcal{P}_{\mathcal{E}}$ than the initial set produces. Thus, upon completion of the first interpolation, we have two estimates with $\boldsymbol{k}^{(v)}$, which are closer to $\mathcal{P}_{\mathcal{E}}$ than the rest, causing the biasing parameters responsible for the smallest estimate to be removed as the 6th data in the second interpolation. Following the same logic, the five sets of biasing parameters used in the last interpolation are the set corresponding to the largest estimate less than $\mathcal{P}_{\mathcal{E}}$, three sets corresponding to $\boldsymbol{k}^{(v)}$, and the initial set. Because the second inflection point is never removed, an exponential interpolant is unable to compute candidates that do not over-perturb the system. The failure would have been avoided if SParSE was configured to either (1) take 4 closest data sets instead of 5, or (2) set the maximum number of interpolations to 4 instead of 3, both of which would have removed the initial set of biasing parameters in the last iteration and yielded viable biasing parameter candidates. Although an additional round of exponential interpolation or decreasing the number of data sets used in it would have prevented the three failures in, we remind the reader that such changes may induce non-convergence in other samples. Lastly, we observed this type of poor agreement between the past data and its exponential interpolant only on over-perturbing initial rates.

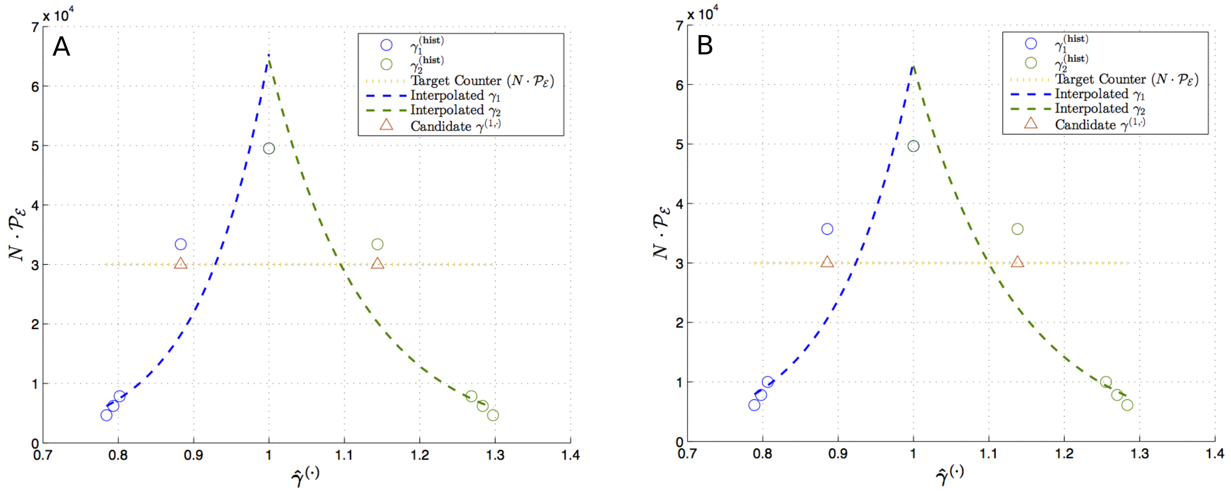

FIG. C.1: Illustration of first exponential interpolation for the two failed initial reaction rates in birth-death process with $\mathcal{P}_{\mathcal{E}} = 0.60$ and $\epsilon_{\mathcal{P}_{\mathcal{E}}} = 0.01$. Figure 1-A represents $\boldsymbol{k}_3^{(0)} = [1.606\ 0.0140]$ and Figure 1-B $\boldsymbol{k}_{27}^{(0)} = [1.684\ 0.0148]$. Yellow horizontal dotted line is the desired number of successful trajectories, *i.e.*, $N \times \mathcal{P}_{\mathcal{E}} = 3 \times 10^4$. Blue and green circles denote the past intermediate biasing parameters for R_1 and R_2, respectively, normalized with respect to $\boldsymbol{k}^{(0)}$. Blue and green dashed lines are the interpolants constructed from the past intermediate biasing parameters, and the red triangles are candidate biasing parameters computed according to Step 3 in Subsection *Updating intermediate reaction rates*.
